# Innovation of Imine Metal Chelates as Corrosion Inhibitors at Different Media: A Collective Study

**DOI:** 10.3390/ijms23169360

**Published:** 2022-08-19

**Authors:** Hany M. Abd El-Lateef, Tarek El-Dabea, Mai M. Khalaf, Ahmed M. Abu-Dief

**Affiliations:** 1 Department of Chemistry, College of Science, King Faisal University, P.O. Box 400, Al-Ahsa 31982, Saudi Arabia; 2 Chemistry Department, Faculty of Science, Sohag University, Sohag 82534, Egypt; 3 Chemistry Department, College of Science, Taibah University, Madinah 344, Saudi Arabia

**Keywords:** corrosion inhibition, metal chelates, potentiodynamic, electrochemical impedance, Langmuir adsorption

## Abstract

The corrosion inhibition of transition metal chelates derived from Schiff base ligands was tested for (mild, copper, stainless, aluminum and carbon) steel in various concentrations of (HCl, HNO_3_ and H_2_SO_4_) acidic medium at 25 °C through (weight loss, potentiodynamic polarization, polarization curves, electrochemical impedance spectroscopy (EIS) and open circuit potential measurements (OCP)) techniques. The studied compounds were identified with various spectral, analytical and physico-chemical techniques. It was observed that the investigated compounds had a significant inhibitory impact on the corrosion of diverse steels in the medium investigated. The analysis shows that increasing the dose of the studied complexes improves the corresponding inhibitory efficiency values. Negative results of Gibb’s free adsorption energy ($\Delta G_{ads}^{0}$) prove the suppression process’s spontaneous and physical adsorption, which contradicts the Langmuir adsorption isotherm. As a result of this insight, a novel bridge between nuclearity driven coordinated inorganic chemistry and materials, as well as corrosion control, has been built. This review provides an overview of the use of Schiff bases and associated transition metals as potential corrosion inhibitors, including the factors that influence their application.

## 1. Introduction

Schiff bases are promising chelating ligands in coordination chemistry due to their propensity to develop stable chelates with transition metals [1,2,3,4,5,6]. Schiff base metal chelates were widely investigated due to their amazing chemical and physical properties. Heterocyclic Schiff bases and their chelates, having N_2_ and O_2_ donor atoms, were investigated by several scientists [7,8,9,10,11,12,13,14,15]. The metal chelates containing (monodentate, bidentate and polydentate) Schiff base ligands have been established and grown in popularity in metal complexes due to their immense binding and structural possibilities, as well as their applications in various fields [8,16,17,18,19,20,21,22]. Scientists have been studying ways to inhibit corrosion damage for many years. Inhibitors could become an efficient tool for slowing down the process of corrosion [23,24]. An effective inhibitor would be an organic molecule possessing sulfur, nitrogen and oxygen atoms that can inhibit metallic corrosion [25,26]. Corrosion is a destructive problem associated with corroded metal through its surroundings. Occurring in the presence of oxygen and humidity on the metal surfaces, chemical corrosion seems to be the uncontrolled degradation of metals induced by heterogeneous chemical reactions. Biochemical corrosion refers to the impact of certain microorganisms on metal [27,28,29]. Corrosion is a redox system in which anode oxidation and cathode reduction take place [30,31]. The damage by corrosion results in one of the most effective and cost-effective tools for the maintenance and defense of materials used [32]. The corrosion process is an interface that comprises the adsorption of chemical compounds on the metal surface. Besides, N_2_ and O_2_ have ligands and their metal chelates have one variety of possible practical importance, including the reduction of metallic loss in engineering components [33,34,35,36,37,38].

The use of corrosion inhibitors is one of the most common ways of corrosion control and prevention. Due to their excellent mechanical properties and inexpensive cost, metals are widely employed as construction materials in most key areas, including in food, petroleum and power production as well as in chemicals and electro-chemical industries, in particular steel [25,26]. The main issue with steel is that it dissolves in acidic media. One of the key areas of concern in many industries where acids are commonly employed for applications such as acid pickling, acid cleaning, acid descaling and oil well acidizing is the corrosion of metals and steel in acidic aqueous solutions. Due to the typical aggressiveness of the acid solution, building materials corrode rapidly. It is critical to include corrosion inhibitors to avoid metal breakdown and reduce acid use. Organic molecules containing N_2_, O_2_ and S atoms are the most well-known acid inhibitors. The organic compounds’ inhibitory activity on the dissolution of metallic species is generally related to adsorption interactions between the inhibitors and the metal surface. A good inhibitor has numerous advantages, including high inhibitory efficacy, cheap cost, minimal toxicity and ease of manufacture. Corrosion inhibitors are chemicals that control, inhibit or prevent metal-corrosive medium reactions. Many effective organic inhibitors contain bonds as well as heteroatoms such as N_2_, O_2_ and S. Schiff bases and metal complexes are among them. A molecule with a lone pair of electrons on nitrogen has been observed to be a good corrosion inhibitor for metals and alloys. Organic compounds with more than one heteroatom-containing electron demonstrate high inhibitory properties in an acidic environment by providing electrons to interact with the metal surface. Other aspects to consider are the molecule’s size, the surroundings and nature of the metal as well as the experimental data including inhibitor concentration, molecular structure and the type of the substituents in the molecule itself. Schiff bases can operate as Lewis bases by donating their lone pair(s) of electrons to the metal, as well as any electrons. The adsorption of Schiff base on the metal surface protects and covers the metal from an aggressive environment, hence inhibiting corrosion [23,24,33,34].

Inhibitors play a critical role in corrosion control by modifying the potentials of corrosive media. Schiff bases are coated on the surface of carbon steel [39,40,41], mild steel [42,43,44,45,46,47], stainless steel [48], copper [49,50], nickel [51], aluminum [52], iron [53] and alloys [54,55] in various violent solutions as a result of the absence of >C=N- groups and operate as an effective corrosion inhibitor by spontaneously producing a special coating and obstructing the active sites on the surface [56,57,58,59,60,61]. Adsorption is how the inhibitors work on the metal surface [62,63]. The performance of inhibitors increases in the arrangement of P > S > N > O, as it depends on the presence of electron density stand out surrounding atoms, the number of presented binding sites for adsorption in a receptor structure size, charge densities, adsorption mechanism and the formation of metal chelates [64,65,66,67]. Different parameters, including the inhibitor’s molecular size, the type of substituent, the nature of the metal and the electrolyte, influence the rate of adsorption on the surface [68]. Quantum chemistry studies that have already shown it to be a highly powerful tool for probing mechanisms have recently described a mechanism of corrosion inhibition [43,44,45]. Density functional theory was used to compute this fundamental chemical structure, which could also aid in the explanation of the inhibition mechanism.

## 2. Overview of Metal Chelates as Corrosion Inhibitors

Lawal et al. (2022) studied the inhibition effect of four REE β-diketone chelates on mild steel and 304 stainless steel in 3.5% NaCl solution, such as cerium hexafluoroacetylacetone Ce(hfac)_3,_ acerium acetylacetone Ce(acac)_3,_ lanthanum hexafluoroacet La(hfac)_3_ and lanthanum acetylacetone La(acac)_3_, and reduced the overall rate of corrosion of mild steel from 156, 166, 180 and 159 µm/y to 42, 41, 43 and 45 µm/y at a concentration of 0.5% of Ce(hfac)_3_, Ce(hfac)_3_, La(acac)_3_ and La(hfac)_3_, respectively [69]. Weight-loss processes and potentiodynamic polarized scans were applied for studying the corrosive environment impacts of the REE β-diketone chelates. The microstructure of mild steel and 304 stainless steel following weight-loss and potentiodynamic studies in 3.5% NaCl solution containing 0.5% weight per volume (*m*/*v*) content of the studied inhibitor was investigated using optical microscopy and SEM. Ft-IR spectra and Raman spectroscopy were also utilized to investigate the type of oxide film which develops on the sample surface under investigation. Surface analysis using Raman spectra indicated the development of a barrier protection film layer on mild steel and 304 SS including a rare earth element oxide and iron oxide/iron oxyhydroxide. As stainless-steel samples were treated to 3.5 percent NaCl, the corrosion rate shifted to more noble values in the existence over all four protective coatings, suggesting that they work as anodic inhibitors. The analyses presented that the four REE β-diketone chelates are very effective corrosion inhibitors of 304 stainless steel and mild steel in a 3.5 percent NaCl solution at temperatures ranging from 20 to 60 °C. As observed, increasing the temperature improved the rate of corrosion, and there was a constant fall in the value for cathodic Tafel coefficients while no inhibitors were applied under the same conditions and environment. The lowering in the cathodic Tafel constant trend proved that the studied REE β-diketone chelates operated as cathodic protective coatings. As a result, the inhibitors are most likely mixed corrosive inhibitors that indicate potency is dependent on nature and the material available when they are applied. The 0.5 percent dose used was insufficient to provide 100 percent inhibitor performance with either the stainless steel (except at ambient temperature in the mass loss studies) or the mild steel, especially as the measuring temperature goes up. It is suggested that a range of higher doses be further studied.

There was one instance of mild evidence that such type of rare earth element steel observed in the corrosion protection may be considerable, but no definitive proof could be assessed that the β-diketone or rare earth element metal played a key role in the corrosion inhibitors results. More research with related inhibitors, for which the β-diketone molecule or rare earth element with corrosion protection is modified, should be conducted in the future [69].

Haruna et al. (2021) studied adsorption isotherms to supply information on how inhibitors interact with the metallic surface [70]. The surface coverage rates at 313 K with various doses were applied to characterize the optimum isotherm models to evaluate the adsorption mechanism. One of the several adsorption isotherms evaluated, the Langmuir isotherm, provided the best fitting, with correlation coefficient (*R*^2^) values close to 1. The regression results for the Langmuir adsorption isotherm for ligand, Mn (II) chelate and Co (II) chelate were, respectively, 0.958, 0.997 and 0.998. Negative $\Delta G_{ads}^{0}$ is compatible with the adsorption system’s spontaneity and the permanence of the adsorbent surface on the Cu surface [71]. $\Delta G_{ads}^{0}$ results of −20 kJ mol^−1^ or below are quite well believed to be linked with the physical adsorption phenomena, which involve the electrostatic attraction of the charged molecule with the charged metals, while that of the −40 kJ/mol or above connected with the chemisorption process [72]. Data of $\Delta G_{ads}^{0}$ for the ligand, Mn (II) chelate and Co (II) chelate are −9.421 KJmol^−1^, −10.445 KJmol^−1^ and −10.379 KJmol^−1^, respectively, showing that the inhibitors are adsorbing physically [68,73].

Jaafar et al. (2020) studied the preparation of thiazine, tetrazole and 1,3-oxazepine Schiff base, and its chelates demonstrated a high level of corrosion protection of mild steel corrosion in acidic media, which may be ascribed to the absence of p electrons in aromatic structures and many bridges enclosing the >C=N- group as well as an electro-negative atom (N) in inhibitor receptor systems. The adsorption mechanism of inhibitory compounds has just been achieved while using the vacant (d) orbital of Fe-atoms as an electron acceptor. As a result, coordinating bonds are formed by overlapping empty 3d orbitals of iron for p orbital electrons in the inhibitor. The effect of the extra hydroxyl functional group on the benzene ring is an inductive effect, resulting in a rise in electronegativity and heterocyclic activation, which would affect more adequate inhibitor absorptivity, enhancing both resistance and adsorption. This implies that corrosion behavior is caused by inhibitor adsorption on the steel exterior and the agent’s performance for the adsorption of the tested inhibitors. Furthermore, it must be indicated that compounds with large sizes and atomic masses could have a role in the increased inhibitory efficiency [74,75]. Figure S1 shows the deterioration of carbon steel in the presence of inhibitors in the optically mild steel image features. In the absence of corrosion protection, the coupon surfaces are hard and oxidized. This corrosion appears to be more prominent in the fusion zone, which has the highest sensitivity to deterioration and may be accountable for high corrosion potential. The image representation of mild steel after corrosion in an acidic media including defenders represents an adherent inhibitor particles’ layer over the metal’s exterior, which protects it [76,77,78].

Ade et al. (2020) studied the inhibition efficiency of 2-[(1H-indol-3-ylmethylene)-amino]-4-methyl-phenol [AMPIA] Schiff base ligand and their Cd (II), Ti (IV), Hg (II) and Zr (IV) metal chelates on carbon steel in three various concentration of acidic medium (0.1, 0.01 and 0.001) and N of HNO_3_, HCl and H_2_SO_4_ by the weight-loss method at room temperature. Under the analysis that the studied compounds have inhibition efficacy, the AMPIA ligand prevents the oxidation of the metal in different acid mediums. Additionally, [Zr(IV)AMPIA], [Ti(IV)AMPIA], [Hg(II)AMPIA] and [Cd(II)AMPIA] metal chelates also exhibit good inhibition efficiency. We find that such inhibitory performance is impacted by the oxidized media as well. Nitric acid is a strong oxidizing agent as compared to the other two mineral acids.

After the analysis of the tested compounds applied for investigation inhibition efficiency, the influence of ligand and their metal chelates is evaluated in Table 1. By inspection of Table 1, we conclude that in 0.1 N, HCl [Cd (II) AMPIA] and [Hg (II) AMPIA] complexes display an inhibition effectiveness of 82.35% and 73.52%, respectively. The ligand shows (Schiff base) inhibition capacity of 42.64%. [Zr (IV) AMPIA] and [Ti (IV) AMPIA] complexes show lower inhibition efficacy. While in 0.01 N HCl solution, the ligand AMPIA shows supreme inhibition efficacy ~88.23% and their [Zr (IV) AMPIA] and [Ti (IV) AMPIA] complexes show lower inhibition efficiency ~60.0%. Instantaneously, [Hg (II) AMPIA] and [Cd (II) AMPIA] complexes display 76.46% and 82.35%, respectively. Inhibition efficiency is associated with the concentration and chemical structure and temperature of the studied compound. The functional groups are responsible for the inhibition of the mild steel corrosion in different acid mediums. The inhibitor property of the studied compounds explains the unlimited protection of mild steel since being in contact with an acidic and corrosive environment. The interesting result obtained from the experiment is that in the HCl acid medium, the studied compounds show better inhibition efficiency than the HNO_3_ and H_2_SO_4_ acid mediums. Thus, the inhibition efficiency was found from more metal chelates and chelating agents and was utilized to study their effect on mild steel corrosion [79].

## 3. Investigation

Tah Murmu et al. (2019) recognized two metal chelates as promising corrosion inhibitors on mild steel, and the variables that influence their operation were identified. The corrosion inhibition in these compounds is a potentially inhibiting effect towards the corrosion of mild steel. Cd (II) and UO_2_ (II) metal chelates were prepared by interacting CdCl_2_·H_2_O and UO_2_ (NO_3_)_2_·6H_2_O with the [N-carbamimidoyl-4-((4-chlorobenzylidene)-amino) benzene sulfonamide] Schiff base ligand in EtOH. They were observed to be effective corrosion regulators for mild steel at a 500 ppm dosage of 1 M Hydrochloric acid solution. Due to their higher size and molecular planarity, the metal complexes appeared to have stronger inhibitory efficacy than free ligands [80]. As a result, the UO_2_ (II) complex has a higher efficiency than the Cd(II) complex.

The weight-loss metho, OCP, EIS and potentiodynamic polarization were used to study the corrosion behavior on mild steel. This demonstrates the corrosion rate of the environment, which is caused by the dissolution of the pre-immersion, air-formed compound coating on the metal’s surface. Due to the inhibitor adsorption on the metal surface, the steady state potential of mild steel moves more toward positive values at varying inhibitor doses. The corrosive density reduces to the extent to which the dosage of its studied complexes increases, and the negative shift in corrosion rate denotes that the studied compounds square measure mixed type relates, but primarily electrode inhibition rather than anodic ones. This occurrence could be related to the presence of a phenyl ring with a high electron density [81]. They may limit metal oxidation in an acid medium due to the adsorption of two metal chelates, Cd^2+^ and UO^2+^. The inclusion of complexes enhanced the inhibitory efficacy of the produced inhibitors, with the optimum attained for the UO_2_ (II) chelate. In 1 M HCl solution, the presence of UO_2_ (II) chelate indicated good corrosion protection for mild steel. This implies that the inhibitor molecules coat the mild steel surface, shielding it well from direct contact with an acidic solution and therefore reducing the metallic solubilization. Since both anodic and cathodic diagrams are moved in the positive direction, the chelates act as a combined type of inhibitor. The inhibitor particles are adsorbed on the metal outers by replacing the surface liquid H_2_O molecules. The adsorption of adsorbates on the electrode surface is an aquasi-substitution activity between inhibitor molecules in the aqueous medium and molecules of water at the surface of the electrode. The observed modes of adsorption (physisorption and chemisorption) could be owing to the examined inhibitor, including several varied molecules, some of which can be adsorbed chemically and others physically [82]. Figure S2 shows the microstructure of the refined sample before corrosion, as well as the deteriorated samples of the blank sample for dosages of 500 ppm. Figure S3 shows SEM images acquired from unexposed and exposed specimen coupons in 1 M HCl solution for one day with a blank sample and 500 ppm for Cd (II) and UO_2_ (II) complexes. The morphology of the mild steel surface indicated the development of a protective surface film that prevents metal dissolving on HCl solution, retards hydrogen evolution and contributes to the mixed-type inhibitory action [83].

Kashyap et al. (2018) studied the corrosion inhibition of compounds under inspection through the impedance spectrum of mild steel in the presence of acidic medium through 100 ppm doses of several TMCSBs, which were also displayed as Nyquist diagrams. EIS characteristics are derived from the impedance spectrum. The Nyquist plot revealed a capacitive loop within the height frequency zone due to the charged transfer resistance (*R_ct_*) and induced loop with little frequency range due to the TMCSB absorption process. Analysis exhibited that (84.19%) MC_2_ was the maximum efficient corrosion inhibitor while related to additionally developed metal chelates. The ordering of protective coatings is TMCSBNi > TMCSBCu > TMCSBCo > TMCSBZn to demonstrate the growth in inhibitor concentration. The enhanced data of *R_ct_* and reduced data of *C*_dl_ (capacitance double layer) of prepared chelates with respect to reference confirm the chelates’ potent inhibition ability. Furthermore, the study found that TMCSB inhibits the corrosion rate for metallic surfaces (mild steel) via an adsorption process. The drop-through *C*_dl_ values could be responsible for the reduction in the limited dielectric standard and/or an improvement in the width of the electrical double layer, signifying that the inhibitor particles are adsorbed at the steel exterior by substituting water molecules [84]. The Nyquist plots are mainly essential for surface quality, solid surface with no uniformity and inhibitor adsorption on metal surfaces. Using Versastudio software, the EIS factors are examined by associating the correct equivalent circuit to the Nyquist diagram. The presence of electrons in a heterocyclic ring, azomethine groups and electron density atoms could explain TMCSB’s corrosion inhibition activity. Furthermore, the methyl group enhances electronegativity and stimulates the aromatic ring over electrostatic interaction, both of which enhance adsorption. These results indicated the TMCSB corrosion behavior is performed by inhibitor adsorption on the metal surface [85,86,87].

Mahross et al. (2017) examined the inhibition efficiency of STSC ligand and (Cu^2+^ and Ni^2+^ and Zn^2+^) chelates for mild steel corrosive environment in oilfield surface water in this study. IEs designed for a mix of 500 ppm STSC and 5 ppm metal ion (Ni^2+^ and Cu^2+^ and Zn^2+^) determined to be 87.96, 88.77 and 85.13%, respectively. Without and with metal ions, the inhibitory efficiency of the STSC ligand and its metal chelates in regulating corrosion for steel in oilfield hydrates were studied. The most efficient corrosion preventive was identified to be STSCCu. Electrochemical methods such as Tafel polarizing and linear and OCP methods were used to acquire the results. Polarization investigations revealed that all employed chelates are anodic inhibitors, as well as the mechanistic features of corrosion behavior. The FT-IR tool was used to examine the passive covering. UV-Visible spectrum data analysis was also employed to detect the existence of the Fe-inhibitor complex. The design of the insoluble compound deposited upon that mild steel surface was related to the corrosion inhibition effect, and the adsorption mechanism reflects the Langmuir adsorption isotherm [86,87,88]. SEM was applied to examine the surface morphology. Figure S4a–c shows SEM images of a bending test immersed in oilfield hydrates for one day without and with an inhibitor system. A slight coating of inhibitory has been discovered on the surface of the metal. Next, the tested compounds were theoretically studied through the DFT (B3LYP) technique with the 6–311G** basis set. Various molecular orbital parameters were determined to aid in the clarification of the inhibition mechanism. The AE statistic has been discovered to be strongly associated with the inhibition efficiency of inhibitors. A relationship diagram was obtained between ΔE and empirical inhibition efficiency (IE percent). Finally, the investigational and simulated results agree well [89].

Kassim et al. (2017) studied the protective abilities of corrosion inhibitors on metals through EIS. This approach does not form a double layer or a solution interface, and it produces consistent results during measurements (Table 2) [90]. EIS tests in (1 M) HCL with and without inhibitor chemicals at 303 K were used to investigate the effect of inhibitor concentration on mild steel. Figure S5 show the Nyquist plot produced with EIS observations on mild steel and (1 M) HCL acid comprising A1 and its various metal chelates, as well as the standard solution. The presence of a single capacitance circuit in the susceptibility spectra with each dose implies that charge transfer occurs on the surface of mild steel. The curves in the resistance graph are not exactly full semicircles, as shown below. It could be due to spectrum dispersion induced by the irregularity of the surface of mild steel [32]. The modest electrical system model is employed to reflect EIS outcomes. Rs, CPE and *R_ct_* are the electrical elements that represent electrolytic impedance, fixed phase element and charge transfer resistance at the layer formed, respectively. Increasing the dosage of inhibitors up to 100 ppm increases *R_ct_* from 119 to 5100 cm^2^. The inhibition efficiency had been 98 percent, demonstrating that thiourea B2 at 100 ppm is an efficient mild steel inhibitor in (1 M) HCl solution. The existence and absence of inhibitors in mild steel in 1 M HCl were measured using the linear polarization resistant (LPR) technology. The electrical and chemical parameters (*E*_corr_), oxidation and reduction Tafel slopes (βc and βa) as well as corrosion density (i_corr_) were determined for the Tafel estimations of curves. The optimum value of reaction rate obtained for A1 during the linear polarization method with various concentration levels is 92%, although for its chelates it is 93% for B1 and 95% for B2. The i_corr_, or electrical field for mild steel, is a type of inhibitor, which was shown to be below that of the blank solution. This demonstrates anticoagulant binding on mild steel [23,24] attributed to the prevalence of heterocyclic groups with high charge distribution, which repressed iron oxidation [91]. Several electrons are transferred to the metal’s unoccupied orbital, resulting in a beneficial process. According to data, the addition of BCTB greatly reduces corrosion potential densities without increasing the corrosion potential (*E*_corr_). The positive improvements in corrosion potential are based on the assumption that the occurrence of inhibitors functions primarily as an electrode surface barrier for mild steel.

It has been demonstrated that the productivity of inhibition with benzoyl thiourea ligand is an optimal association through the addition of inhibitor doses. Due to their adhesion to an interface of mild steel in acidic conditions, all of the examined complexes and ligands exhibit higher polarization resistance. Since B2 ([N-(benzylcarbamothioyl) benzamide] nickel(II) acetate) complex have extra possible sides to be bound on mild steel, their inhibitory effectiveness is larger than that of A1 (N-(benzylcarbamothioyl) benzamide) and B1 ([N-(benzylcarbamothioyl) benzamide] copper(II) acetate). Many factors influence inhibition efficacy, including adsorption sites, mechanism of contact, molecule size and inhibitor shape. The reduced size of the B1 complex compared to the B2 complex may account for its lower efficiency. As a result, the inhibitory efficacy of the investigated chemicals varied as A1 < B1 < B2 (Table 2) [92].

Veni et al. (2017) investigated the influence of produced ligands and their metal chelates on mild steel corrosion in (0.1 M) HNO_3_, and weight-loss studies for mild steel were performed at RT. The degree of inhibition performance and corrosion inhibition was determined through weight control after 2 days. According to the statistics, the studied compounds demonstrated significant corrosion inhibitory activities on mild steel corrosion in an (0.1 M) HNO_3_ environment that is utilized as an oxidant for corrosive environment. The Schiff base ligand, as well as its Co(II) and Cu(II) metal chelates, were employed to investigate the inhibitory performance. The growth inhibition performance data show that both combinations are effective inhibitors [108] and the inhibitory efficiency of the Schiff base towards steel material could be related to co-ordination through donor–acceptor links between unbounded electron pairs of the ligand and metal donor atoms. The metal complexes inhibited more effectively than the free ligand. The metal complexes’ increased effectiveness relative to the Schiff base might be related to greater mass and structural morphology [109]. As a result, the order of effectiveness is L < [CuL] < [CoL] [93].

Das et al. (2017) investigated the corrosion-preventing qualities of the produced cadmium Schiff base complexes in HCl solution. The Schiff base ligand L1 [N, N-dimethyl-N′-(1-pyridin-2-yl-ethylidene)-ethane-1,2-diamine] was used initially, followed by the production of three cadmium complexes. The corrosion control ability of Cd(II) chelates was well investigated. The [Cd(L1)_2_](ClO_4_)_2_ complex, in the absence of a co-ligand, was not shown to be particularly potent in corrosion protection. Furthermore, no increase in corrosion inhibition property was detected after using cyanoacetic acid as a co-ligand in [Cd(L1)(Cyanoacetate)(OAc) complex, and this could be due to its monomeric character and lower amount of adsorbing sites. To enhance the surface active site, azide ion was inserted as a co-ligand in [Cd_2_(L1)_2_(N_3_)_4_] complex and structurally adsorbing site was enlarged, as was the nuclearity of the complex due to the connecting nature of the co-ligand. According to the findings, the azido bond dimeric complex [Cd_2_(L1)_2_(N_3_)_4_] demonstrated a major corrosion prevention property against mild steel. Considering the development of azide ion’s potential as a co-ligand for inhibitory properties, we considered using the other Schiff base ligands L2 [2-morpholino-N-(1-(pyridin-2-yl) ethylidene)ethanamine] and [(2-(piperidin-1-yl)-N-(1-(pyridin-2-yl)ethylidene)ethanamine] L3 for the formation of well-designed polyimide complexes [Cd(L2)(N_3_)_2_]n and [Cd_2_(L3)_2_(N_3_)_4_]n with very high corrosion inhibitor qualities attributed to the prevalence of a growing incidence of binding sites, so this elevated inhibition performance compares favorably to other inorganic systems [110,111] and organic inhibitors [112]. *R_ct_* organized the corrosion development on mild steel, according to the semi-circular Nyquist plot for an unconstrained solution. Rp, including all resistances among metal and solution surfaces, was involved in this case. Moreover, in the case of more complex systems, Bode graphs might provide more information. Figure S6 shows the Bode graphs of the produced inhibitors. To compare the resistance to corrosion of various samples. Figure S7 shows the phase angle maps on mild steel, with and without inhibitors in (15%) HCl. These also support the conclusions drawn from the Nyquist and Bode graphs. ElS, weight-loss studies and potentiodynamic polarization have all demonstrated that azide compounds exhibit corrosion inhibitory properties. SEM images indicate which cadmium complexes protected the mild steel surface. These tests were carried out after the (OCP) had been stabilized [113]. Seeing the similar phenomenon related to the behavior of various particles extracted from multiple investigational and theoretical results, it is reasonable to infer that growing nuclearity is a major component in improving resistance to corrosion polymer matrices metal chelates, which merits further examination. The increased efficiency of complex [Cd(L2)(N_3_)_2_]n could be attributed to the fact that it efficiently coated the metal surface, delaying mild steel disintegration [95,113,114].

Baboukani et al. (2016) studied the polarization curves of 316 L stainless steel on several investigated compound doses in a sulphuric acid solution. As displayed in Figure S8, the electrical and chemical parameters Icorr and Ecorr as well as βc and βa were estimated. It demonstrates that the value of Ecorr determined using the sulphuric acid solution is pushed to the optimal side. The result implies that this molecule is mostly adhesive on the surface’s anodic sites. A study of data revealed that, in comparison to the template, an increase in dosage of Co complex resulted in continuous growth in cathodic density, so adding Co chelate up to 200 ppm does not change the pathway of this mechanism [115]. Co complex works as a mixed-type inhibitor, including having a strong influence on the anodic process. At the minimum tested doses (50 ppm), Co complex suppresses Fe dissolution in the anodic while accelerating hydrogen evolution in the cathodic reaction. Increasing the insertion of Co complex causes the anodic sections to be displaced to poor corrosion current density, whereas the cathodic section stays relatively unharmed. EIM of 316 L SS in (0.1 M) the sulphuric acid solution, during the existence of multiple dosages, is seen in Figure S8b. The obtained EIS exhibits a depressed sensitive cycle at high frequencies (HF), prompted by an attractive cycle at the low-frequency range (LF). The conductive impedance of the surface oxide films and the solution enveloped in between the electrode surface or the positive electrode is represented by the crossing point of the conductive loop with of real axis. *R_ct_* and Rs reflects the charge-transfer permeability, with which the value is a test of electron transmission through the surface and is thus inversely related to the corrosion degree [116]. To achieve an exact match, CPE is used in the loop rather than a pure dual designed [117]. The electrical and chemical statistics are derived by combining the observed EIS values with the corresponding circuit. *IE* percent of the tested inhibitors was computed using the equation [118]:

(1) $$IE/\%=\left[\frac{R_{ct}^{i}-R_{ct}^{0}}{R_{ct}^{i}}\right]\times 100$$

where $R_{ct}^{0}$ and $R_{ct}^{i}$ denote the charge-transfer contact resistance without and with the inhibitors, respectively. Table 3 shows that the presence of Co chelate improves the related *IE* percent value until C_inh_ = 100 ppm. Figure S8b also shows that the specific frequency of the inductance cycle reduces as the amplitude of such electrode resistivity increases, regardless of the adsorbent dose. Experiencing restricting film inhibits charge carrier pathways to low-frequency range in resistive Nyquist plots. The input impedance EIS data have been matched onto the only one-time constant corresponding circuit with the induction loops moved under the investigated lower frequencies barrier. The inclusion of the tested compounds improves the inductive looping diameter of the Nyquist diagrams without modifying their unique properties, according to data analysis. This suggests that the inhibition of inhibitor is related to the adsorption upon that steel surface without changing the corrosion process. Impedance measurements verified the results of the polarization experiments. Adsorption behavior can be applied to study the mechanism of corrosion protection. The modeling and prediction of straight lines with a slope greater than one demonstrate that the tested compounds are adsorbed following Langmuir’s isotherm [94].

Nassar et al. (2015) synthesized and characterized new Zn(II), Ni(II), Co(II) and Cu(II) chelates with Bis(di-acetylmonoxime)biphenyl-3,3′-dimethoxy-4,4′-diamine Schiff base (H_2_L) through physicochemical and spectroscopic methods. The corrosion inhibition for the studied compounds on mild steel surface in (0.5 M) hydrochloric acid was conducted employing weight loss, potential dynamics polarization and scanning optical microscope. The effect of substrate concentrations on inhibition proficiency in the absence of various doses of the tested compounds reveals which (7 × 10^−4^) Mol/dm^3^ in (0.5 M) HCl solution provides the best inhibitory activity. The Co(II) complex had a good influence on corrosion behavior. Due to the rising molecular mass of molecules, the sequence of inhibitory effectiveness follows the pattern 4 < 1< 5 < 3 < 2. The increase in carbon steel inhibition efficiency in (0.5 M) HCl solution with a growing dose can be described by associative adsorption. It was also found that increasing the temperature induced the coating film to partially degrade and remove itself from the carbon steel surface [119]. Compound adsorption was discovered to reflect the Langmuir adsorption isotherm concept with mixed type inhibition performance. In addition, the optical images of the carbon steel surface in Figure S9a reveal carbon steel degradation with inhibitors. The decomposition is more visible near the surface layer because these locations are more disposed to corrosion and could be in control with the increasing degree of corrosion. The optical microscope scans demonstrated that the absorption process of the studied compounds on the metal surface preserved the surface in the HCl medium Figure S9b–f. Finally, the prepared compounds could be employed as corrosion inhibitors. The polarization studies’ data are in agreement with the results gained from the weight-loss approach. It should also be noted that the high size and high molar mass could contribute to improved inhibitory efficacy and the tested compounds prompted both (anodic and cathodic) reactions. As a result, those particles might be categorized as (anodic or cathodic) inhibitors [64,96].

Abd El-Lateef et al. (2015) described the synthesis and characterization of HBSAP and HBSAQ Schiff bases. The structures of the tested compounds were studied and investigated using single crystal X-ray diffraction and CHN and spectra techniques (UV-Vis and NMR and IR). The inhibition activity on carbon steel in the (0.1) M HCl + 3.5% NaCl solution for all long and short immersion time was planned using electrochemical and theoretical studies as well as surface characterization. The inhibiting performance improves as the inhibitor dosage increases and decreases as the process temperature rises. This indicates that as more inhibitor particles are coated upon the metal surface, leading to a greater surface exposure, the molecules operate as high adsorption inhibitors [120]. The potentiodynamic polarization demonstrates that the inhibitor molecule is now more immobilized in cathodic areas. Its performance rises as inhibitors concentrations rise (92.8 percent at the ideal dose of (10^−3^ M) for HBSAQ). This means that the hydrogen growth and metal dissolution were both inhibited.

**Figure 1:**
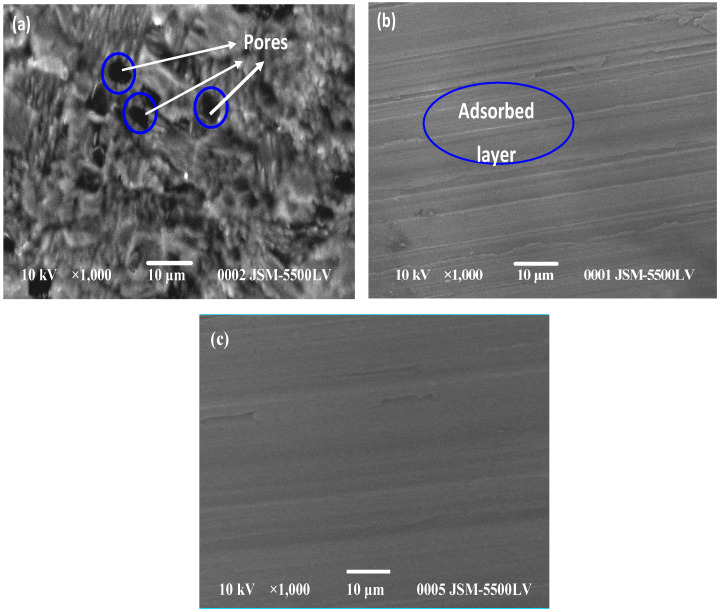
SEM studies of carbon steel in (3.5% NaCl + 0.1 HCl) solutions (**a**) without inhibitor during 2 days immersion and (**b**) with (10^−3^ M) of HBSAQ during 2 days and (**c**) with (10^−3^ M) of HBSAQ during 10 days immersion. Reproduced from the permission of Ref. [97].

Moreover, the parallel modification of the anodic and cathodic Tafel curves demonstrates that the inhibition process in the absence of the studied inhibitors is activated and mediated, but the electrochemical corrosion mechanism is stable with Schiff base inhibitors [121]. The EIS data confirmed that as the inhibitor dose increased, the *R_ct_* was enhanced and the double layer permeability decreased. The inhibition performance improves significantly with an increasing immersion time of 120 h and then remains stable from 120 to 240 h. This can be related to the development of a strong inhibitor layer that inhibits corrosive media from attacking the surface of the metal. Moreover, the data indicate that the inhibition performance has been maintained over 120 h of immersion. The inhibitors’ adsorption on the surface of activated carbon was revealed to obey Langmuir’s adsorption behavior via a strong mixing physicochemical interaction with the activated carbon surface, as demonstrated through SEM. SEM analysis supports the adsorbed of HBSAQ or the modification of the activated carbon surface depicted in Figure 1, which is highly corroded through the corrosion medium, giving in a rough, porous and heterogeneous layer of reaction yields through a grain structure. The inhibitor was found adsorbed on the binding sites of carbon steel, resulting in an improved surface. According to data, the studied Schiff base compounds are highly effective carbon steel inhibitors in the studied aggressive solution, as the power of preservation already achieved 95.2% at 240 h of immersion. Furthermore, the data on the corrosion inhibition of the surface of the metal were performed to validate the agreement with the electronic structure simulations utilizing quantum chemistry techniques [97].

Abd El-Lateef et al. (2015) applied CHN and ^1^H NMR and X-ray, IR and ^13^C NMR, and UV-Vis spectroscopy to prepare and structurally analyze HNMA and DMSMA. A series of processes, including potentiodynamic polarization, EIS, SEM, EDX and theoretical results, were used to evaluate their inhibiting performance on mild steel in an (0.5 M) H_2_SO_4_ solution. 

**Figure 2:**
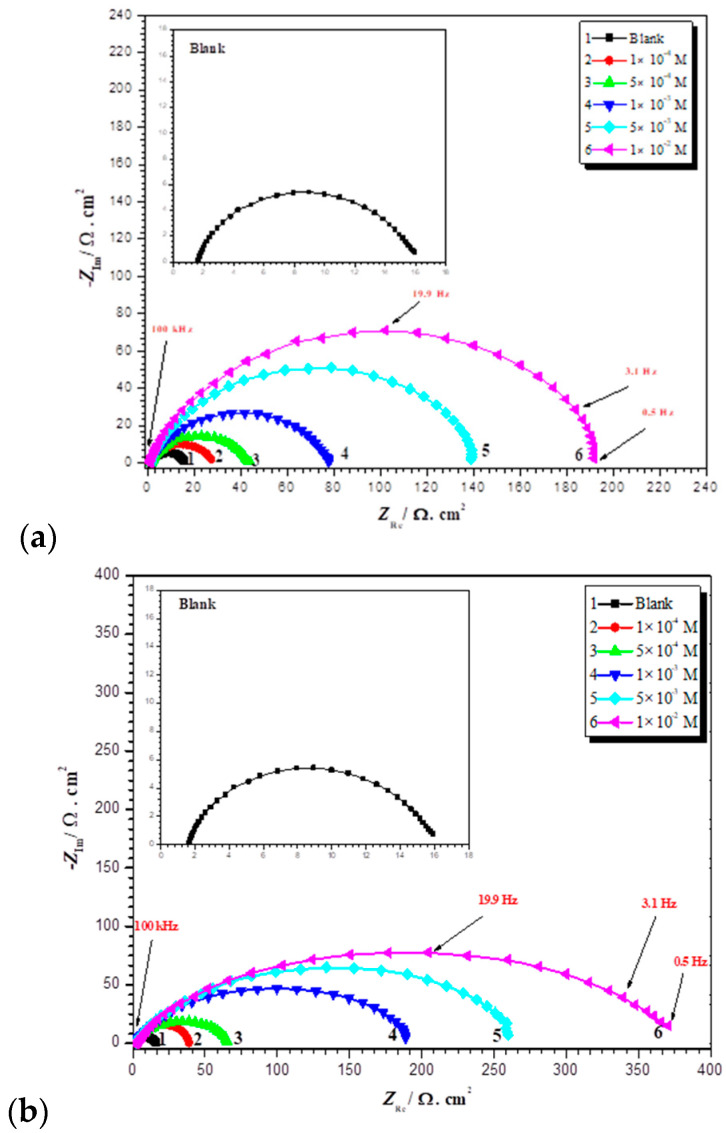
Nyquist diagram for mild steel in (0.5 M) H_2_SO_4_ at 50 °C with and without different doses of (**a**) HNMA and (**b**) DMSMA inhibitor. Reproduced with the permission of Ref. [98].

**Figure 3:**
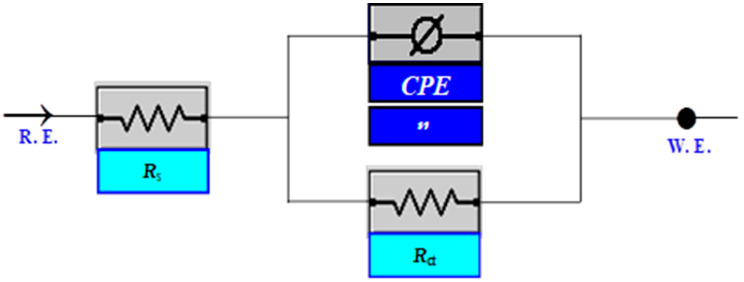
With and without inhibitors, an equivalent circuit model is utilized to fit impedance data. (*R*_s_ stands for solution resistance, *R_ct_* is for charge transfer resistance and CPE stands for constant phase element). Reproduced with the permission of Ref. [98].

The results demonstrate that HNMA and DMSMA are inhibited well, and their inhibition efficacy rises with concentration, obtaining an optimum of 92.14% and 96.1% at 1.0 mM of HNMA and DMSMA, respectively. The polarization results indicate that the studied compounds were used as mixed inhibitors. Figure 2 shows the Nyquist plots for mild steel in 0.5 M H_2_SO_4_ at 50 °C with the individual inhibitors HNMA and DMSMA. The addition of HNMA and DMSMA resulted in a large increase in total impedance. Additionally, the inductance reaction of mild steel altered considerably in the presence of tested inhibitors in the 0.5 M H_2_SO_4_ solution, due to a rise in electrode resistance as inhibitors concentrations rise. The semicircular shape of the Nyquist plot indicates that the CT process occurs during metal breakdown [122]. The impedance of the different Nyquist plots is examined through fitting results to a simple form, as shown in Figure 3. Impedance tests showed that the mild steel dissolution was primarily controlled by a CT mechanism in the absence and presence of the studied inhibitors. The adsorption of HNMA and DMSMA on the surfaces of metal reflects the Langmuir adsorption isotherm. The inhibitor compounds form a well-protecting coating on the steel surface, according to SEM micrographs. Quantum chemical results show a relation between restrictive abilities and molecular parameters. Furthermore, the theoretical study provided illuminating explanations for the inhibitory mechanism [98].

**Figure 4:**
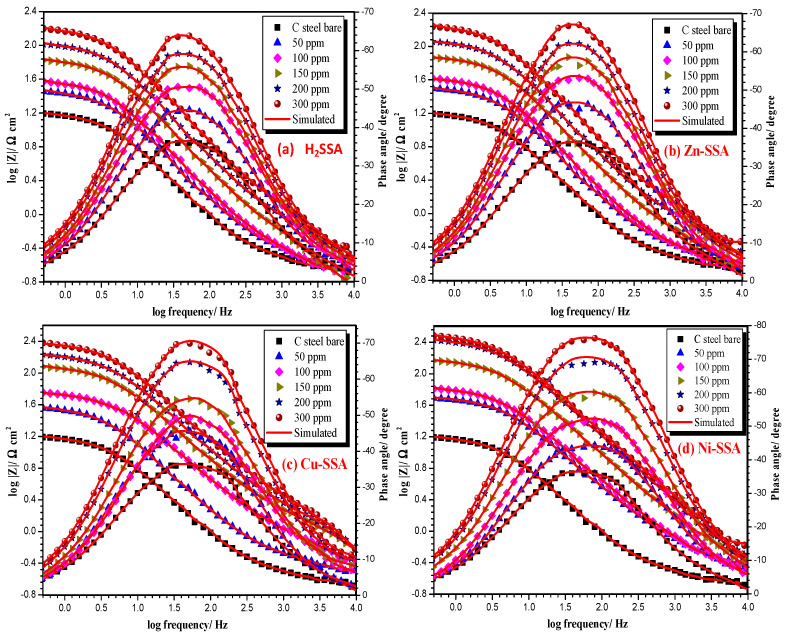
Phase angle and Bode diagrams for CS electrode in (1.0 M) hydrochloric acid with and without various doses of the studied inhibitors (**a**) H2SSA, (**b**) Zn-SSA, (**c**) Cu-SSA and (**d**) Ni-SSA at 50 °C. Reproduced with the permission of Ref. [99].

Abd El-Lateef et al. (2018) synthesized three polar (Cu-SSA, Ni-SSA and Zn-SSA) metal chelates of H_2_SSA ligand. The tested compounds were characterized with various physicochemical techniques. The inhibition performance of studied H_2_SSA and M-SSA on the carbon steel corrosion (CS) in HCl (1.0 M) was estimated through various electrochemical tools (Table 2). The inhibition activity increased by increasing the inhibitor dose. The data obtained from EIS and PDP technique exhibited that H_2_SSA and their metal chelates act as (active and mixed) inhibitors. The adsorption of the tested compounds on the CS surface obeyed the Langmuir isotherm paradigm. The Bode phase angle charts (Figure 4a–d) identify a single optimum around various frequencies, with the optimum extending inside the inhibitor-containing solution, which would be related to the growth of such a protected film on the surface of the CS electrode. The impact of resistance in the presence of inhibitors is greater than in the absence of inhibitors, and the value of resistance increases with the increasing inhibitor dose. SEM/EDX tests indicated the formation of an outer covering that shields the CS surface from direct corrosion attack by the adsorbed inhibitor molecules on the CS surface. The amount of corrosion products reduces as the peaks associated with salts and oxides generated during the corrosion process are decreased. Furthermore, DFT analysis was used on the empirical observations. The obtained theoretical results are highly compatible with the inhibitory actual data [99].

**Figure 5:**
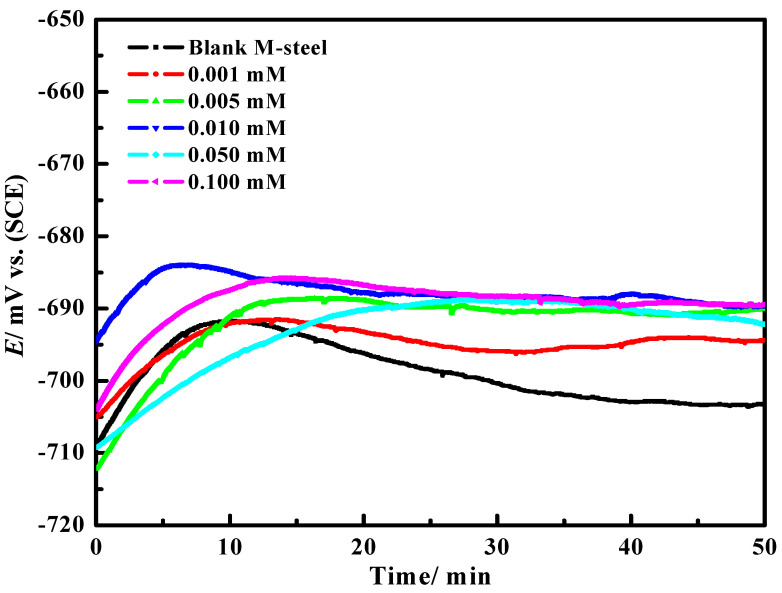
E_ocp_ vs. time diagram for mild steel electrode immersed in (CO_2_-3.5% NaCl), with and without different concentrations of VOPHL at 50 °C. Reproduced with the permission of Ref. [100].

**Figure 6:**
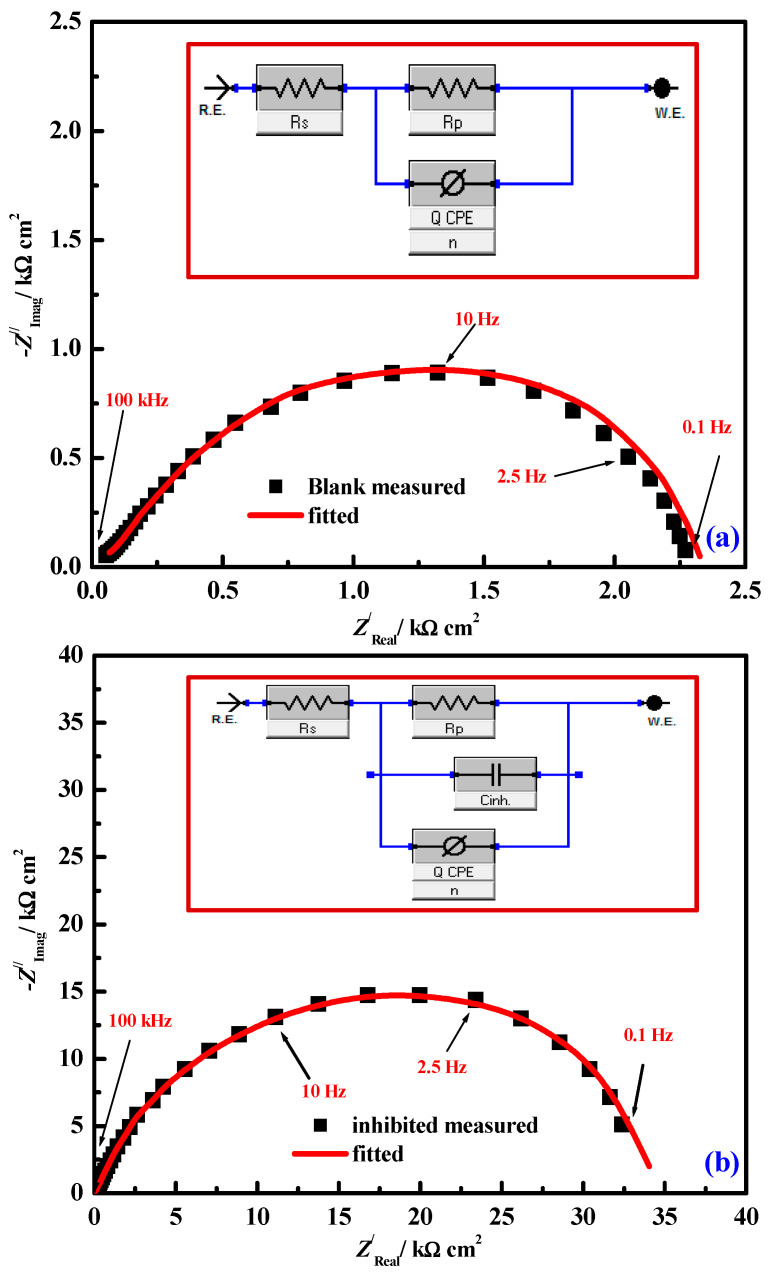
Comparison of empirical EIS results (black points) estimated for M-steel substrates immersed in CO_2_-saturated brine and the simulated (red line) for some of the data shown (**a**) the uninhibited solution and (**b**) inhibited solution in the presence of 0.1 mM VOPHL. Inset equivalent electric circuit used in the fitting of EIS in the blank (inset (**a**)) and inhibited (inset (**b**)). Reproduced with the permission of Ref. [100].

Adam et al. (2020) prepared two VOPHL and NiPHL chelates by chelation of H_2_PHL ligand with VO(II) or Ni(II). The structures are fully estimated through different spectral tools. The anticorrosion studies using mild steel, H_2_PHL, VOPHL and NiPHL indicated fundamental protection action using mild steel in NaCl solutions saturated through CO_2_ which was studied using PDP and EIS. PDP studies display that H_2_PHL, NiPHL or VOPHL operate mixed-type inhibitors, i.e., well prevents both (cathodic and anodic) processes through the chemical adsorption on the surface of CS that obeys a Langmuir model. To estimate the ideal exposure time for EIS and PDP, Eocp-t study was applied. Figure 5 shows the change of E_ocp_ using time per minute for the mild steel electrode immersed in CO_2_-3.5% NaCl solution with and without various doses of VOPHL at 50 °C. All graphs related to the inhibited process showed equal properties through the detected period for study. Later, the steady-state assessment for E*_ocp_* proficiency up to 50 min of immersion was the ideal contact range for electrochemical analyses. Finally, the immersion time and the E_ocp_ data trended to stabilize and mentioning adsorption and desorption of inhibitor compounds extended a dynamic balance. Data exhibited that NiPHL and VOPHL display supplementary inhibition effectiveness in comparison to their relating ligand. H_2_PHL, NiPHL and VOPHL exhibited the maximum inhibition protection within 89.57, 97.25 and 98.22%, respectively. The resulting Nyquist diagrams are fixed through Z-view software to achieve the suitable corresponding circuit as exposed through Figure 6a,b in the reference and inhibited medium, respectively. The corresponding equivalent circuit (EQC) utilized for examining the EIS results is displayed in Figure 6a,b (inset). All semi-circles exhibited a considerably dejected nature through a center below the real axis, associated with the scattering as a result of different physical phenomena, with roughness and in-homogeneity of the electrode through corrosion systems. SEM/EDS studied the development of a shielding layer upon the mild steel surface of the inhibiting reagent. DFT results were applied to the tested compounds and were close to the investigational inhibitory possibilities. Theoretical data are in good agreement with corrosive effects [100].

**Figure 7:**
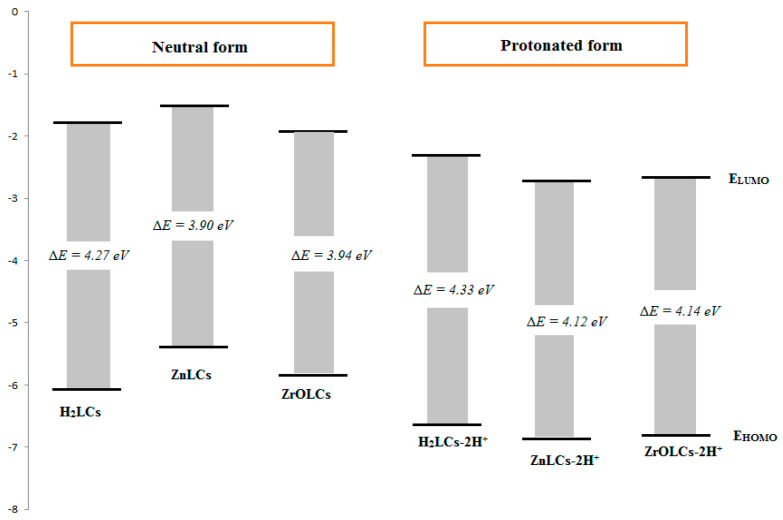
LUMO and HOMO energies of neutral and protonated designs of the inhibitors. Reproduced with the permission of Ref. [101].

**Figure 8:**
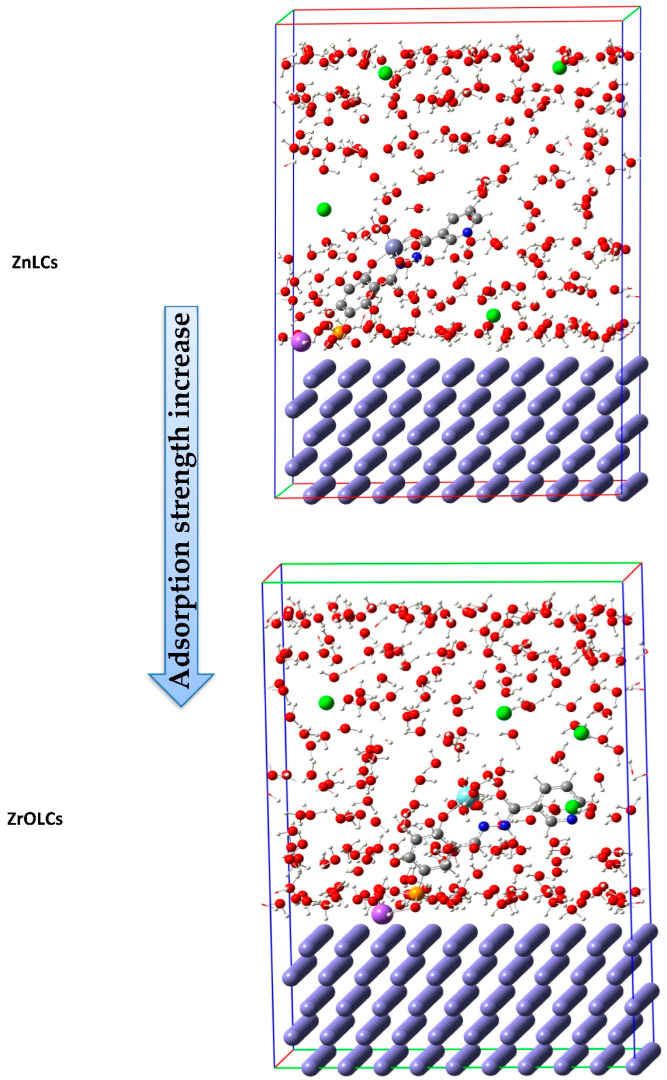
Symbolic photos of ZrOLCs and ZnLCs on iron (110)/250 H_2_O + 4H_3_O^+^ + 4Cl^−^ systems. Reproduced with the permission of Ref. [101].

Abd El-Lateef et al. (2021) prepared and fully characterized H_2_LCs ligand and their corresponding ZnLCs and ZrOLCs chelates. Inhibition performances of the tested H_2_LCs ligand and its complexes for M-steel in a (1.0 M) HCl solution were estimated through (EOCP vs. time and PDP and EIS and FE-SEM/EDX and FT-IR and theoretical approaches (DFT and MC simulations)) techniques. The PDP studies exhibited that all studied ligand and their chelates performed as mixed-type inhibitors and adsorbed on M-steel surface through chemisorption and physisorption, resulting in the Langmuir diagram model. H_2_LCs, ZrOLCs and ZnLCs display enhanced protection capacities, preventing the serious deterioration of ZrOLCs and ZnLCs, at 97.4% and 93.5%, at 5 × 10^−4^ mol/L and at 303 K, respectively. FT-IR and FE-SEM/EDX analyses found a coating film of the tested inhibitors molded on an M-steel electrode. To develop a better ability for adsorption of molecule species on the steel interface, both DFT and MC simulations protected the empirical results that display ZnLCs and ZrOLCs as good inhibitors. The QSAR model was also inspected through the multiple linear regression techniques. That association between the protonated and neutral systems of the data obtained and displayed in Figure 7, exhibited that the results of energy of HOMO orbital of the protonated designs are lesser than neutral designs representing the probability of fewer charitable electrons through protonated designs, as well as the energy of LUMO orbital drops that directs an enhancement in electron acceptance features. Data of the energy gap of neutral designs are lower than in the protonated designs representing a growth through adsorption of neutral designs upon the iron surface. The absorption of neutral design is further adsorbed then protonated, because the energy gap is lesser in the neutral design. As exhibited in Figure 8, ZnLCs and ZrOLCs chelates adhesion perpendicular upon Fe surface. The inhibitor compound is steadied through SO_3_Na group near the surface of Fe. The principal negative result of adsorption energy, signifying greater performance and effective adsorption and the order of shield ability for the tested compounds, is as follows: H_2_LCs < ZnLCs < ZrOLCs. The present study delivers very significant outcomes in designing and scheming viable inhibitors for M-steel in an acidic medium with high protection performance [101].

**Figure 9:**
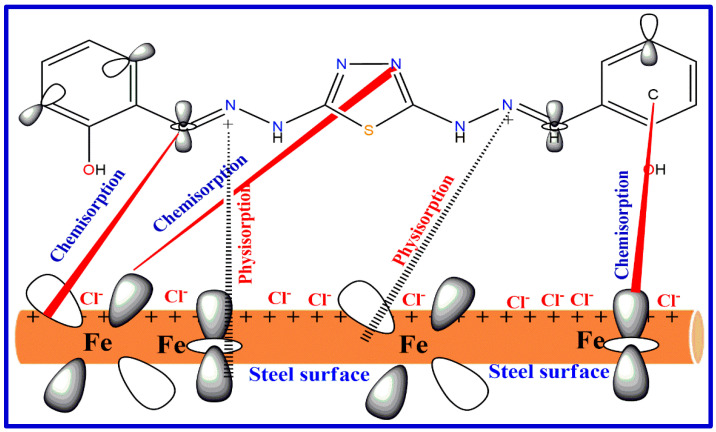
The proposed adsorption model of the imine molecule on steel surface in acidic chloride solution.

Abd El-Lateef et al. (2022) synthesized two novel azomethine chelates for iron (III) from two (NABS and MABS) dibasic tetra-dentate chelating ligands. The tested structures were characterized using various physicochemical and spectral tools. The tested compounds were observed as anticorrosive layers of CS in the HCl medium through theoretical and electrochemical studies such as PAP, EIS and PDP. The inhibition performance is augmented with the increase in inhibitor concentration. The tested compounds displayed protection ability between 84.5 and 96.8% in the existence of 0.5 mmol/L. The PDP technique approves the studied particles displayed as mixed inhibitors. The Langmuir model is used by tested inhibitors since it includes both physisorption and chemisorption adsorption on the metal interface. Figure 9 shows the schematic representation of adsorption of the imine molecules on the steel surface by the chemical attraction between unshared electron pairs of imine molecule and the empty d-orbitals of iron metal ions and electrostatic attraction between the negatively charged of the steel surface (which produces from the adsorbed negative anions such as Cl^−^) and the (N^+^), protonated -NH^+^ groups. The SEM studies were applied to study the surface morphology with and without inhibitors. The surface morphology of the substrate was scanned through SEM. The results obtained through electrochemical and SEM techniques prove the corrosive management ability of the tested particles on the steel contact. The studied particles displayed good efficacy towards pitting corrosion of carbon steel through shifting the pitting potential to nobler affinity. The tested compounds were examined as pitting additives for the CS degradation in a 1.0 N hydrochloric solution with or without various doses of NABSFe. Figure S10A displays the potentiodynamic anodic polarization (PAP) diagram for CS corrosion in a 1.0 N hydrochloric solution including different doses of NABSFe at a stretch rate of 0.2 mV/s. Figure S10B displays the relationship among *E*_p_ and log C_inh_ of parsley oil. A linear line was obtained, indicating that as the concentricity of the NABSFe increases, the stability of the active layer upsurges and the *E*_p_ values shifts into a positive trend. This explains that CS resistance to opposing aggressivity increased and the NABSFe is classified as opposing corrosion enhancers. As a good agreement between experimental methods and corresponding DFT, the observed data from DFT displayed which NABSFe chelates offer the best protective effects [102].

## 4. Conclusions and Outlook

This review focuses on using Schiff base ligands and their metal chelates as significant corrosion inhibitors for (mild, copper, stainless, aluminum and carbon) steel in various concentrations of HCl, HNO_3_ and H_2_SO_4_ acidic medium at 25 °C through weight loss, potentiodynamic polarization, polarization curves, OCP and EIS techniques, and the factors that govern their use. A great variety of these tested compounds containing hetero atoms show remarkable corrosion inhibition activity which has opened the field of research. Most of the studied compounds reported here are effective corrosion inhibitors for steel in acidic media. The higher the inhibition efficiency, the higher the adhesion of adsorbates on steel and the reduction of the corrosive environment. All of the molecules discovered are anodic inhibitors. The corrosion inhibition effectiveness is determined through corrosive media, acid type, acid concentration, PH, inhibitor type and inhibitor interaction with the metal. The usage of tested compounds as metal corrosion inhibitors is limited and very promising. Negative results of Gibb’s free adsorption energy ($\Delta G_{ads}^{0}$) demonstrated the spontaneous suppression mechanisms, physical adsorption and specific surface area of Cu were investigated using the SEM technique. The SEM revealed that the tested inhibitors adsorb to the metal’s surface.

According to the literature, Schiff base metal chelates are generally utilized as inhibitors for mild steel and carbon steel alloys; thus, its usage as corrosion resistant species for all other metals and alloys, particularly copper and aluminum, should be investigated. Conventional Schiff bases, which are manufactured using typical hazardous amines, cannot be employed as ecofriendly corrosion inhibitors due to rising ecological sustainability issues. As a result, the use of bio-derived amines such as chitosan and glucosamine is strongly promoted. A review of the literature reveals that lately only a few publications have been published describing the corrosion prevention performance of chitosan-based Schiff bases. However, their synthesis and application in this context must be investigated further. These substances are environmentally friendly due to their natural and biological origins. Overall, most of the Schiff bases and their metal chelates operate as mixed-type corrosion inhibitors, slowing both Tafel reactions. SBs can be observed to act as interface-type corrosion inhibitors in EIS investigations. Their adsorption was mostly governed by the Langmuir adsorption isotherm model. The literature on the research also suggests that a correct balance of hydrophilicity and hydrophobicity is required for the effective corrosion prevention of Schiff base metal chelates, as too much hydrophobicity reduces their solubility in polar electrolytes. It was also discovered that the majority of Schiff bases and metal chelates have been investigated as corrosion inhibitors in less aggressive conditions. As a result, their application in industrial processes, such as acid pickling, acid descaling, oil-well acidification and acid cleaning that uses extremely acidic solutions, such as 15–28 percent HCl and H_2_SO_4_ solutions, should be investigated.

## Figures and Tables

**Table 1:** Inhibition effectiveness of (AMPIA) Schiff base ligand and their chelates on corrosion in three various concentrations of acidic medium (0.1, 0.01 and 0.001) N of HNO_3_, HCl and H_2_SO_4_ [79].

Compound	AMPIA	[Cd(II)AMPIA]	[Ti(IV)AMPIA]	[Hg(II)AMPIA]	[Zr(IV)AMPIA]
**I.E. (%)**	42.64	82.35	19.11	73.52	14.70
**0.1 N HCl**					
**I.E. (%)**	88.23	82.35	11.76	76.47	64.70
**0.01 N HCl**					
**I.E. (%)**	50.00	75.00	83.30	83.30	75.00
**0.001 N HCl**					
**I.E. (%)**	29.03	45.16	12.90	22.58	16.12
**0.1 N HNO_3_**					
**I.E. (%)**	50.00	75	50	66.66	41.66
**0.01 N HNO_3_**					
**I.E. (%)**	60.00	60.00	40.00	40.00	20.00
**0.001 N HNO_3_**					
**I.E. (%)**	61.11	75.92	7.40	81.48	11.11
**0.1 N H_2_SO_4_**					
**I.E. (%)**	37.5	37.5	37.5	12.5	25
**0.01 N H_2_SO_4_**					
**I.E. (%)**	66.66	66.66	33.33	66.66	16.66
**in 0.001 N H_2_SO_4_**					

**Table 2:** Summarizes the study assessed in terms of compounds, media and metals analyzed.

No	Compounds	Media	Metal	Ref No.
1	2-hydroxy-benzoic acid [1-(2-hydroxy-phenyl)-propylidene]-hydrazide. (H_2_hbpp) *	1.0 M HCl	Mild steel	[90]
	H_2_hbpp Mn(II) complex *			
	H_2_hbpp Cu(II) complex *			
	H_2_hbpp Zn(II) complex *			
2	N-(benzylcarbamothioyl)benzamide (A1) *	1 M HCl	Mild steel	[92]
	[N-(benzylcarbamothioyl)benzamide] copper(II) acetate(B1) *			
	[N-(benzylcarbamothioyl) benzamide] nickel(II) acetate (B2) *			
3	Schiff base Ligand derived from p-chlorobenzldehyde and	0.1 M HNO_3_	Mild steel	[93]
	o-amino phenol. (L)			
	Cu(II) complex. (CuL) *			
	Co(II) complex. (CoL) *			
4	Co-phenanthroline. (PhCo) *	0.1 M H_2_SO_4_	316 L stainless steel	[94]
5	Thiazine Schiff base ligand and [Ni (II), Co (II), Cu (II) and Hg (II)] Metal Complexes. (3) *	0.1 M HCl	Mild steel	[78]
	tetrazole Schiff base ligand and [Ni (II), Co (II), Cu (II) and Hg (II)] Metal Complexes. (4) *			
	1,3-oxazepine Schiff base ligand and [Ni (II), Co (II), Cu (II) and Hg (II)] Metal Complexes. (5, 6) *			
6	1-{(Z)-[(2-hydroxyphenyl) imino]methyl}naphthalen-2-ol Schiff base ligand. (L) *	0.1 M HCl	Copper metal surface	[73]
	1-{(Z)-[(2-hydroxyphenyl) imino]methyl}naphthalen-2-ol and Mn (II) Complex. (MnL_2_) *			
	1-{(Z)-[(2-hydroxyphenyl) imino]methyl}naphthalen-2-ol and Co (II) Complex. (CoL_2_) *			
7	[N,N-dimethyl-N′-(1-pyridin-2-yl-ethylidene)-ethane1,2-diamine] (L1). *	15% HCl	Mild steel	[95]
	[2-morpholino-N-(1-(pyridin-2-yl)ethylidene) ethanamine] (L2) *			
	[(2-(piperidin-1-yl)-N-(1-(pyridin-2-yl)ethylidene) ethanamine)] (L3) *			
	[Cd(L1)_2_](ClO_4_)_2_ *			
	[Cd(L1)(cyanoacetate)(OAc)] *			
	[Cd_2_(L1)_2_(N_3_)_4_] *			
	[Cd(L2)(N_3_)_2_]_n_ *			
	[Cd_2_(L3)_2_(N_3_)_4_]_n_ *			
8	N-carbamimidoyl-4-((4-chlorobenzylidene)-amino) benzenesulfonamid Schiff base ligand *	1 M HCl	Mild steel	[83]
	Cd(II) complex *			
	UO_2_ (II) complex *			
9	Salicylaldehyde thiosemicarbazone Schiff base ligand (STSC) *	Oilfield formation	Mild steel	[89]
	STSC Cu(II) complex *			
	STSC Ni(II) complex *			
	STSC Zn(II) complex *			
10	2-[(1H-indol-3-ylmethylene)-amino]-4-methyl-phenol (AMPIA) *	(0.1 and 0.01 and 0.001) N (HCl and HNO_3_ and H_2_SO_4_)	Carbon and mild steel	[79]
	[Ti(IV)AMPIA] complex *			
	[Zr(IV)AMPIA] complex *			
	[Cd(II)AMPIA] complex *			
	[Hg(II)AMPIA] complex *			
11	(E)-4-(3-Hydroxybenzylideneamino)-2,3-dimethyl-1-phenyl-1,2-dihydropyrazol-5-one (Intermediate) schiff base ligand (TMCSB) *	1.0 M HCl	Mild steel	[85]
	Zinc metal complex (MC1): TMCSBZn *			
	Nickel metal complex (MC2): TMCSBNi *			
	Cobalt metal complex (MC3): TMCSBCo *			
	Copper metal complex (MC4): TMCSBCu *			
12	cerium acetylacetone. Ce(acac)_3_ *	3.5% NaCl solution	Mild steel and 304 stainless	[69]
	cerium hexafluoroacetylacetone. Ce(hfac)_3_ *			
	lanthanum acetylacetone. La(acac)_3_ *			
	lanthanum hexafluoroacetylacetone. La(hfac)_3_ *			
13	Bis(di-acetylmonoxime)biphenyl-3,30-dimethoxy-4,40-diamine *	0.5 M HCl	Mild steel	[96]
	Co_2_L(H_2_O)_2_(Cl)_2_·2H_2_O *			
	Ni_2_L(H_2_O)_2_(Cl)_2_·2H_2_O *			
	Cu_2_L(H_2_O)_2_(Cl)_2_·2H_2_O *			
	Zn_2_L(H_2_O)_2_(Cl)_2_·2H_2_O *			
14	5-bromo-2-[(E)-(pyridin-3-ylimino)methyl]phenol (HBSAP) *	3.5% NaCl + 0.1 M HCl	carbon steel in	[97]
	5-bromo-2-[(E)-(quinolin-8-ylimino)methyl]phenol (HBSAQ) *			
15	1-{(Z)-[(3,5dimethylphenyl) imino]methyl}naphthalen-2-ol (HNMA) *	0.5 M H_2_SO_4_	mild steel in	[98]
	5-(diethylamino)-2-{(Z)-[(3,5-dimethylphenyl)imino] methyl} phenol (DMSMA), *			
16	H_2_SSA (2-[(2-Hydroxy-5-sodium sulfonate-benzylidene)-amino]-benzoate) *	1.0 M HCl	carbon steel corrosion (CS)	[99]
	(Cu-SSA and Ni-SSA and Zn-SSA) *			
17	terephthaloyl salicylidene dihydrazone (H_2_PHL) *	3.5% NaCl in NaCl	Mild steel	[100]
	VOPHL and NiPHL *		C-steel	
18	5-sodium sulfonate-2-hydroxybenzylidene)nicotinohydrazone (H_2_LCs) *	1.0 M HCl	Mild steel	[101]
	(ZnLCs) and (ZrOLCs) *			
19	2,2′-((1E,1’E)-((4-nitro-1,2-phenylene)bis(azanylylidene))bis(methanylylidene))bis(4-bromophenol) (NABS) *	1.0 N HCl	Carbon steel	[102]
	2,2′-((1E,1’E)-((4,5-dimethyl-1,2-phenylene)bis(azanylylidene))bis(methanylylidene))bis(4-bromophenol) (MABS). *			
20	1-{(Z)-[(2-hydroxyphenyl)imino]methyl} naphthalen-2-ol *	0.5 M H_2_SO_4_	Carbon steel	[103]
	Bis-phenanthroline chloro copper (II) chloride di-para-aminobenzoic acid tetrahydrate complex *			
	[Cu(Phen)_2_Cl]Cl (pABz)_2_·4H_2_O (CuPAB)			
21	L-histidine (L_1_) and (L_2_) Schiff base ligands *	2.0 M H_2_SO_4_	Aluminum steel	[104]
	[CuL_1_·EtOH] and [CuL_2_·EtOH] *			
	[NiL_1_·(H_2_O)_3_] and [NiL_2_·EtOH] *			
	[CoL_1_·(H_2_O)_3_] and [CoL_2_·H_2_O] *			
22	4-Chloro-2-(2-oxo-1, 2-dihydro-indol-3-ylidene amino)-benzoic acid Schiff base (ACBAI) *	0.1 NHNO_3_	Mild steel	[105]
	[Ti(IV) ACBAI] complex *			
	[Zr(IV) ACBAI] complex *			
	[Cd(II) ACBAI] complex *			
	[Hg(II) ACBAI] complex *			
23	furfuraldehyde and 4,5-dimethyl-1,2-phenylendiamine (L1) *	1 M HCl	(410 and 304) stainless steel	[106]
	furfuraldehyde and 4,5-dichloro-1,2-phenylendiamine (L2) *			
	[ZnL_1_](AcO)_2_·H_2_O and [ZnL_2_](AcO)_2_·H_2_O *			
	[PdL_1_]Cl_2_ and [PdL_2_]Cl_2_ *			
24	Hapdhba *	0.5 M HCl	Copper steel	[107]
	cis-[Mo_2_O_5_(Hapdhba)_2_]·H_2_O *			
	trans-[UO_2_(Hapdhba)_2_] *			
	[Pd(Hapdhba)Cl(H_2_O)]·H_2_O *			
	[Pd(bpy)(Hapdhba)]Cl·H_2_O *			
	[Ag(bpy)(Hapdhba)]: *			
	[Ru(Hapdhba)_2_(H_2_O)2]·2H_2_O *			
	[Rh(Hapdhba)_2_(H_2_O)Cl]·3H_2_O *			
	[Au(Hpadhba)Cl_2_]·H_2_O *			

**Table 3:** EI parameters of 316 L SS in 0.1 M sulphuric acid solution with and without different dosages [94].

Inhibitor	Blank		Co Complex	
***C*_inh_ (ppm)**	0	50	100	200
***R_ct_* (Ω)**	124.40	407.40	490.80	480.60
**CPE-T (F)**	1.1 × 10^−4^	7.4 × 10^−5^	7.8 × 10^−5^	9.4 × 10^−5^
**IE%**	-	68.90	74.40	73.80
***R*_s_ (Ω)**	2.36	1.98	1.90	1.77

## Data Availability

The raw/processed data generated in this work are available upon request from the corresponding author.

## Supplementary Materials

The following supporting information can be downloaded at: https://www.mdpi.com/article/10.3390/ijms23169360/s1.
