# Supplementary material for: Innovation of Imine Metal Chelates as Corrosion Inhibitors at Different Media: A Collective Study
Source: Int J Mol Sci. 2022 Aug 19;23(16):9360. doi: 10.3390/ijms23169360 (PMC9409127; doi:10.3390/ijms23169360)
Supplement: Supplementary file 1 [file ijms-23-09360-s001.zip › ijms-1817515-supplementary.pdf]

International Journal of
Molecular Sciences

MDPI

Supporting Data

Innovation of Imine Metal Chelates as Corrosion Inhibitors at Different Media: A Collective Study

Hany M. Abd El-Lateef [1,2,*], Tarek El-Dabea[2], Mai M. Khalaf [1,2] and Ahmed M. Abu-Dief [2,3,*]

[1]Department of Chemistry, College of Science, King Faisal University, P.O. Box 400 Al-Ahsa 31982,

Saudi Arabia

[2]Chemistry Department, Faculty of Science, Sohag University, Sohag-82534, Egypt

[3]Chemistry Department, College of Science, Taibah University, Madinah, P.O. Box 344, Saudi Arabia

* Correspondence: authors: hmahmed@kfu.edu.sa, hany_shubra@science.sohag.edu.eg (H. M. Abd El-Lateef), amamohammed@taibahu.edu.sa; ahmed_benzoic@yahoo.com (A. M. Abu-Dief).

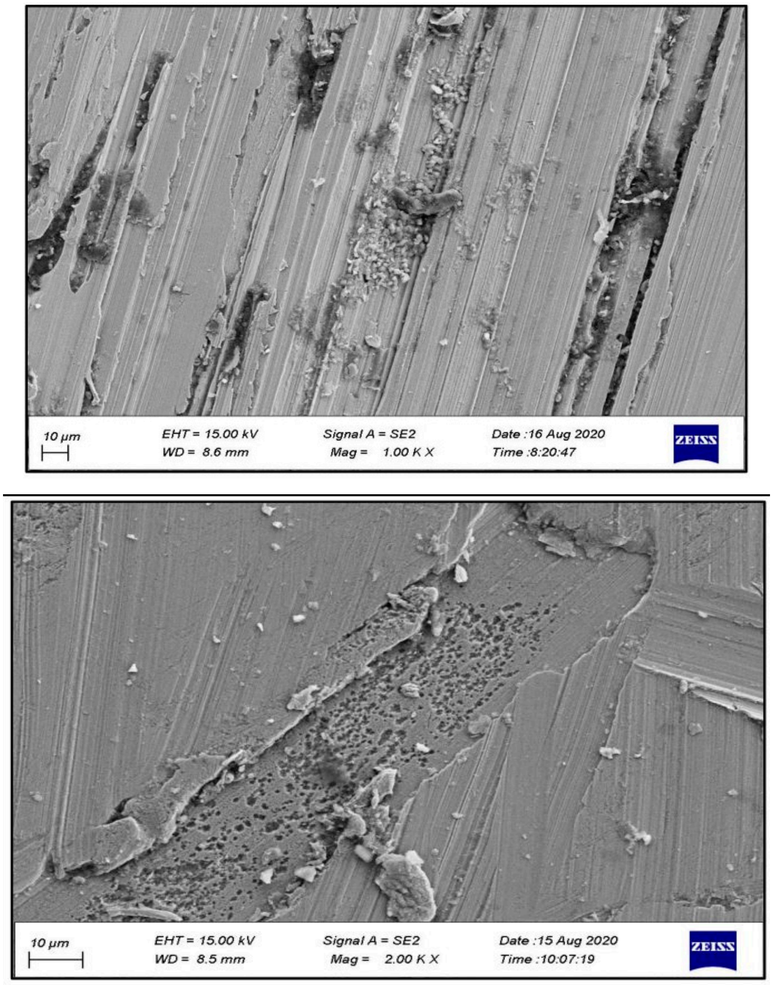

Figure. S1: Mild steel sample photos in acidic solutions without inhibitor. [57].

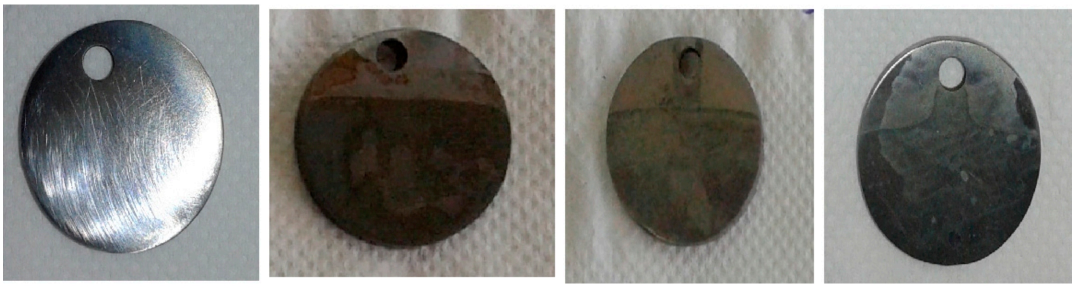

Figure S2: The mild steel macrostructure for the standard sample and the examined complexes [Cd(II) and UO2(II)]. [62]

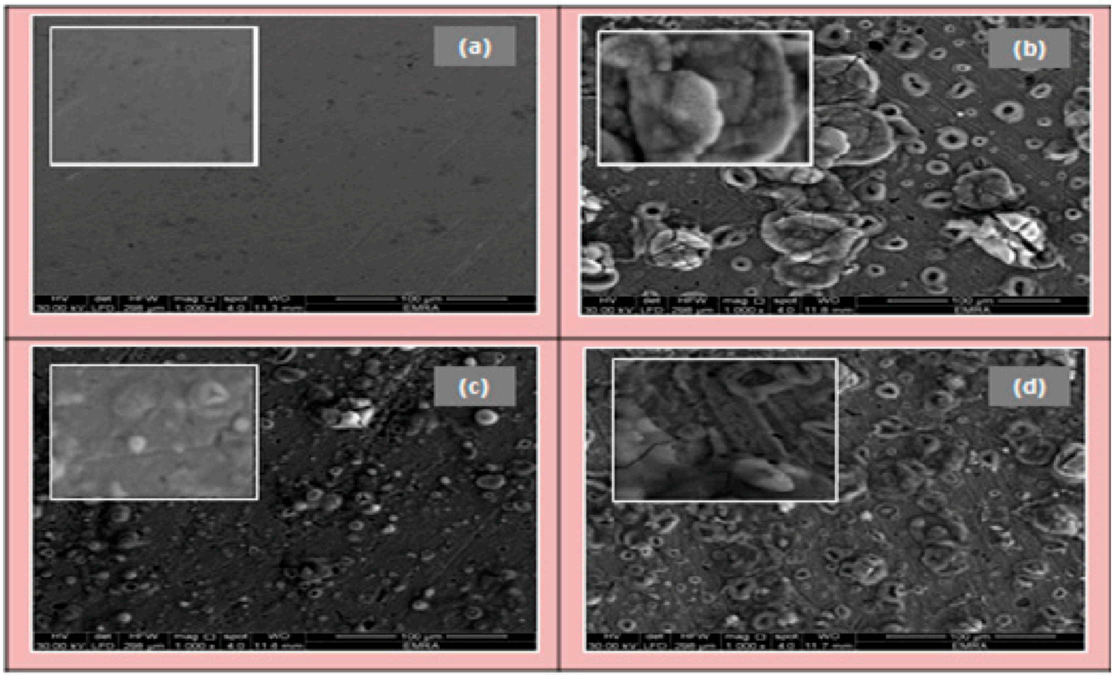

Figure S3: Micrographs of mild steel samples for a glossy mild steel surface that has not been treated (a), in the presence of inhibitor, following absorption in 1 M HCl solution (b), following absorption in 1 M HCl solution containing 500 ppm Cd(II) chelate (c)and through absorption in a 1 M HCl solution with 500 ppm UO_2(II) chelate (d). [62]

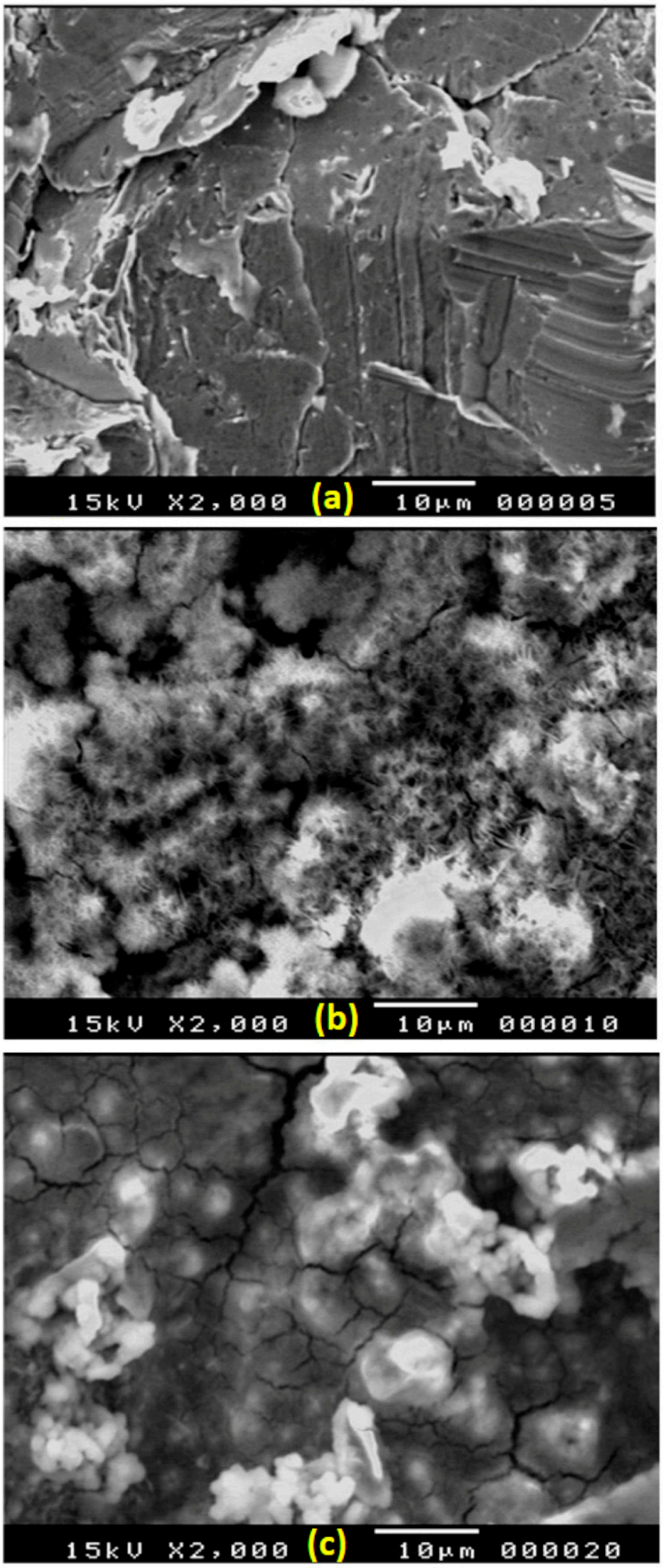

Figure S4: SEM studies of: (a) refined mild steel, (b) oilfield formation water absorbed mild steel (Blank) & (c) mild steel in oilfield formation water + 500 ppm STSC + 5 ppm Cu^{2+}. [67]

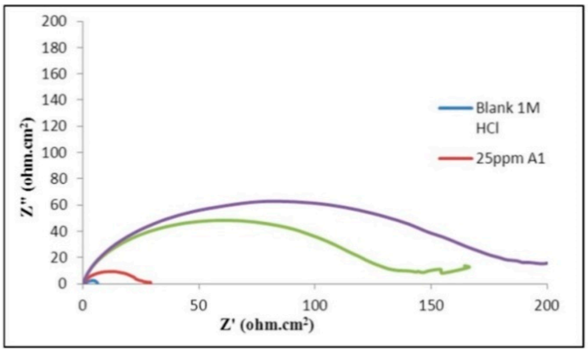

(a)

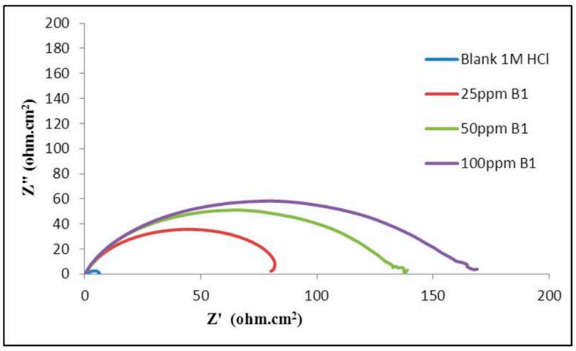

(b)

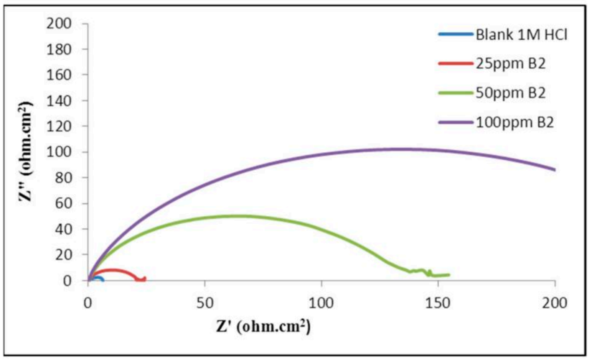

(c)

Figure. S5: Nyquist charts of mild steel corrosion in 1M Hydrochloric acid with and without the various concentrations levels of (a) A1 [N- (benzylcarbamothioyl) benzamide**],** (b) B1 [N- (benzylcarbamothioyl) benzamide] copper(II) acetate & (c) B2 [N-(benzylcarbamothioyl) benzamide] nickel(II) acetate. **[70]**

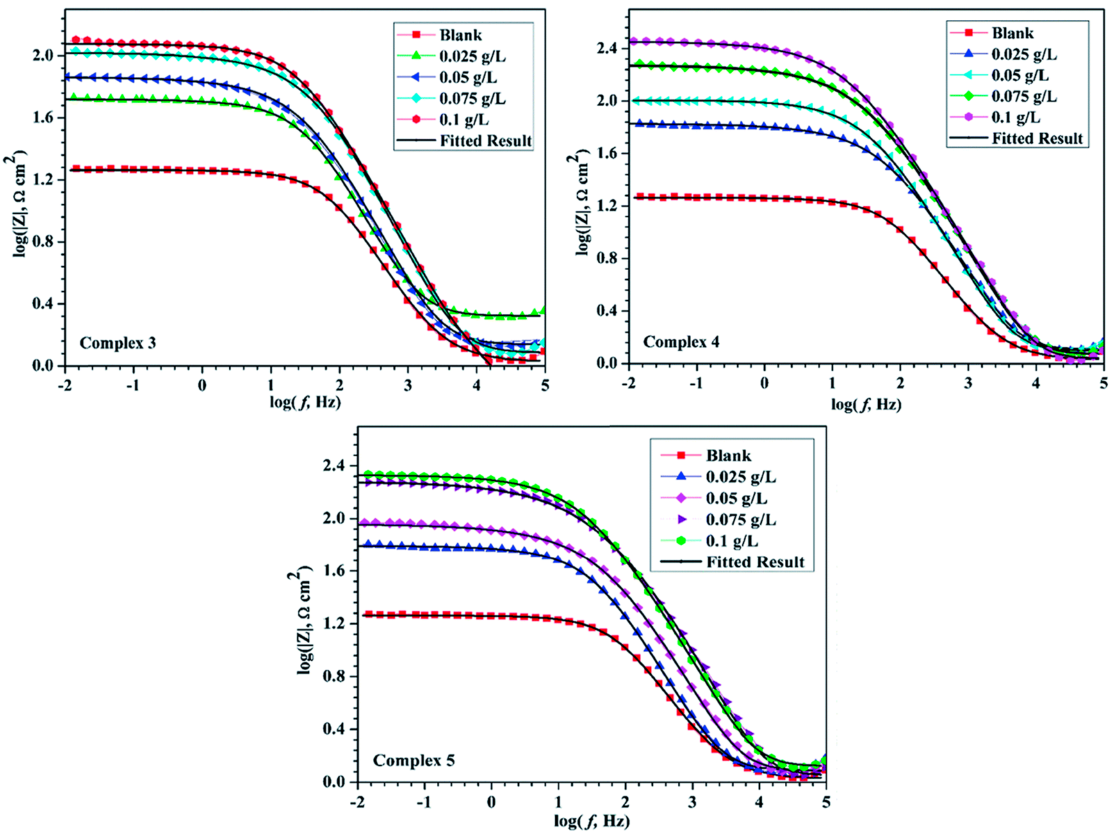

Figure. S6: Bode chart of mild steel in 15% Hydrochloric acid in absence and presence of inhibitor doses. [78]

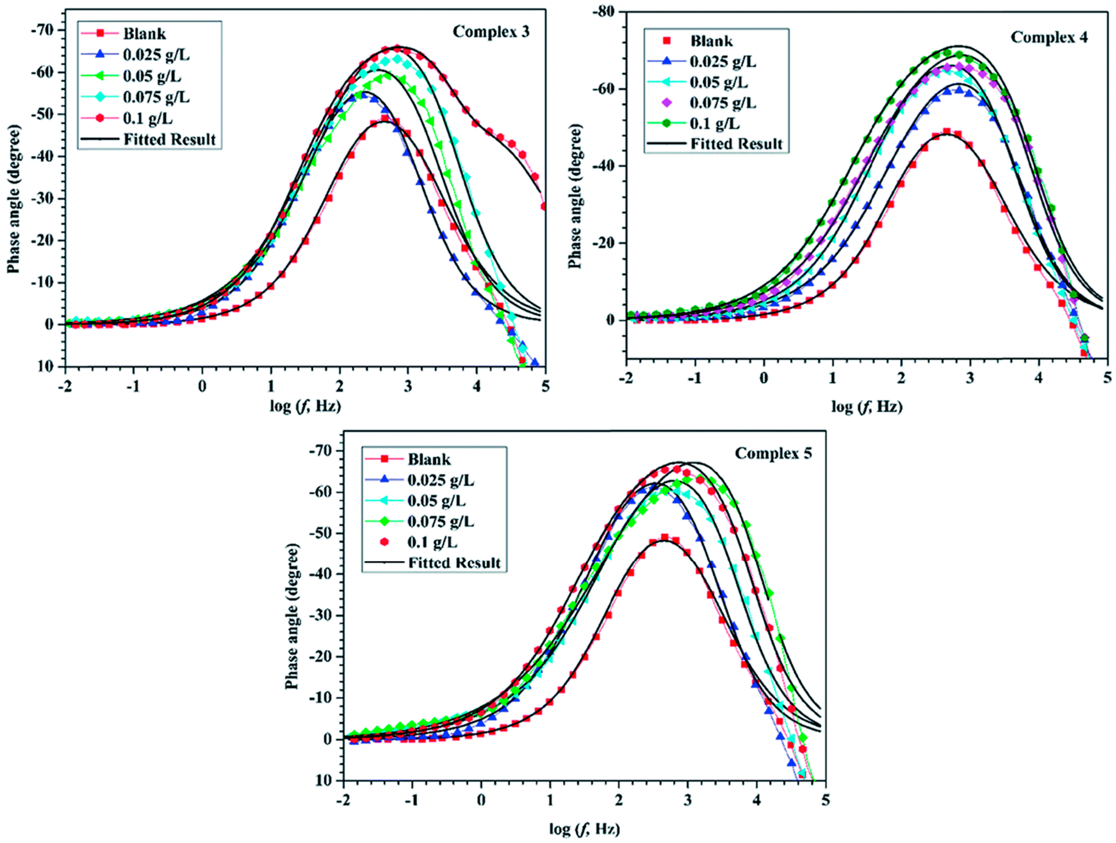

Figure. S7: Plots of phase angles of mild steel in 15% Hydrochloric acid in absence and presence varied inhibitor doses. [78]

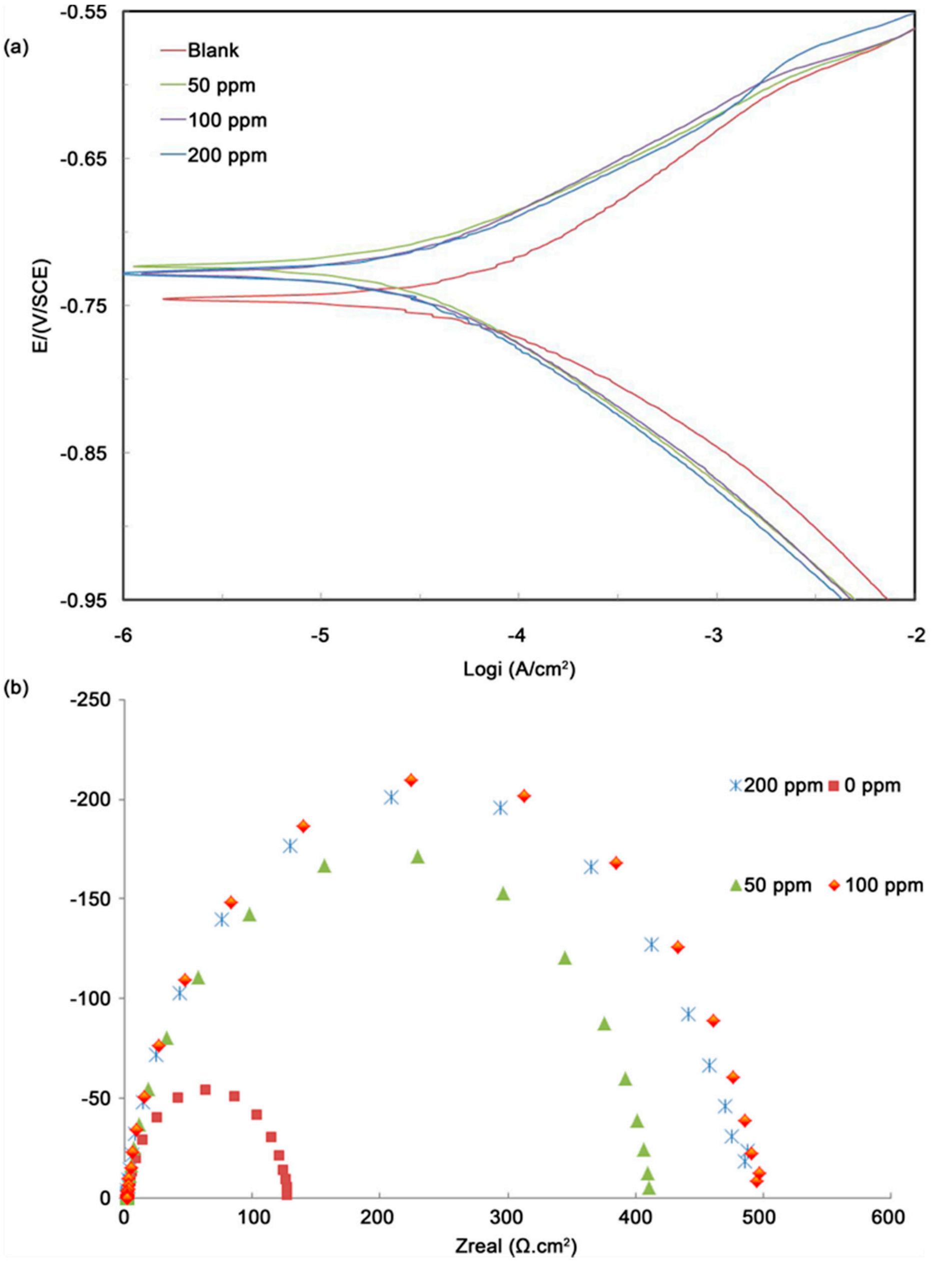

Figure. S8: (a) Polarization curves and (b) Nyquist plot of 316 L S S in 0.1 M Sulphuric acid solution with and without different dosages. [84]

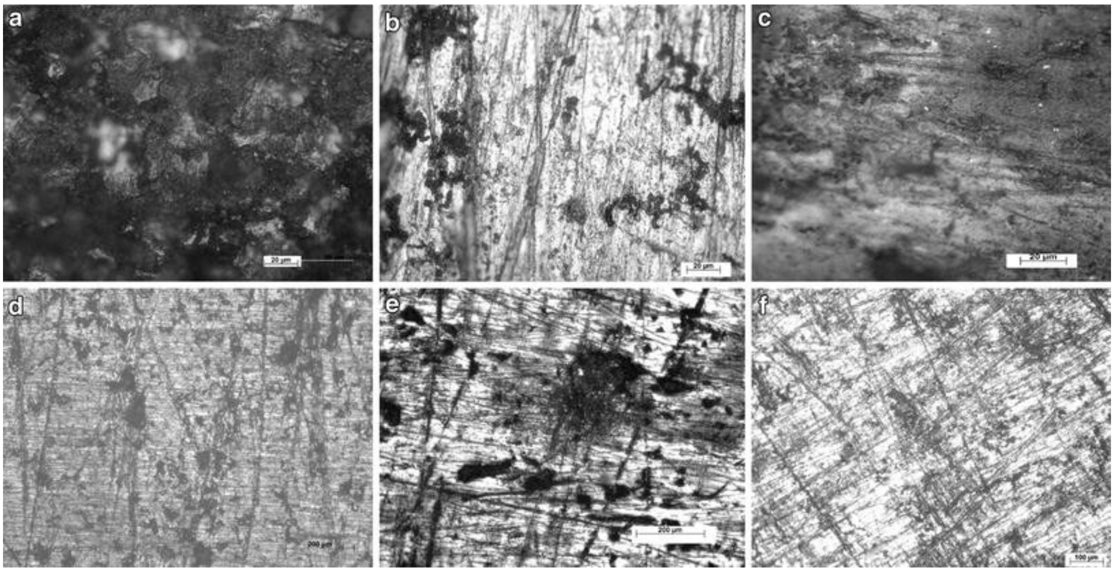

Figure. S9: Optical microscope images of carbon steel sample in acid medium without inhibitor (a), and with inhibitors H2L (b)& Co2L(H2O)2(Cl)2.2H2O (c), Ni2L(H2O)2(Cl)2.2H2O (d), Cu2L(H2O)2(Cl)2.2H2O (e), and Zn2L(H2O)2(Cl)2.2H2O (f).[86]

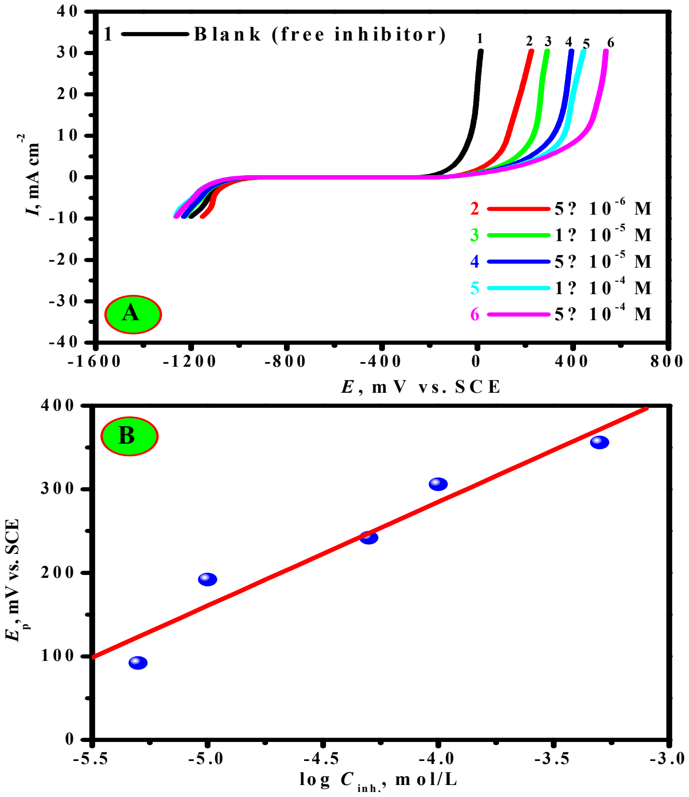

Figure. S10: PAP digrams of carbon steel in 1.0 N HCl solutions with and without various doses of NABSFe at 30 °C (A) and a relation between Ep & logCinh (B) [95]

References

[64] Nassar, A. M., Hassan, A. M., & Shoeib, M. A. Synthesis, characterization and anticorrosion studies of new homobimetallic Co (II), Ni (II), Cu (II), and Zn (II) Schiff base complexes. J Bio Tribo Corros, **2015**, 1(3), 1-16.

[78] Jaafar, W. A., & Saeed, R. S. Synthesis, characterization and corrosion inhibition study of new heterocyclic compounds and schiff base with [Co (II), Ni (II), Cu (II) and Hg (II)] complexes. Syst. Rev. Pharm, **2020**, 11(10), 134-143.

[83] Taha, R. H., Gaber, G. A., Mohamed, L. Z., & Ghanem, W. A. Corrosion inhibition of two schiff base complexes on the mild steel in 1M HCl solution. Egy J Chem, **2019**, 62(1), 367-381.

[88] Mahross, M. H., Efil, K., El-Nasr, T. A., & Abbas, O. A. Experimental and Theoretical Study on Corrosion Inhibition of Mild Steel in Oilfield Formation Water Using Some Schiff Base Metal Complexes. J Elect Sci Tech, **2017**, 8(3), 222-235.

[91] Kassim, K., Nordin, N. A., Zulkifli, N. Z., & Hashim, N. Z. N. Synthesis of Ni (II) and Cu (II) complexes with N-(benzylcarbamothioyl) benzamide as corrosion inhibitors for mild steel in 1M HCl. In AIP Con Proc, **2017**, 1904(1), 020024.

[93] Baboukani, A. R., Sharifi, E., Akhavan, S., & Saatchi, A. Co complexes as a corrosion inhibitor for 316 l stainless steel in H2SO4 solution. J Mat Sci Chem Eng, **2016**, 4(9), 28-35.

[94] Das, M., Biswas, A., Kundu, B. K., Mobin, S. M., Udayabhanu, G., & Mukhopadhyay, S. (). Targeted synthesis of cadmium (II) Schiff base complexes towards corrosion inhibition on mild steel. RSC advances, **2017**, 7(77), 48569-48585.

[101] Abd El-Lateef, H. M., Desoky M. Mohamad, A., Shehata, M. R., & Abu-Dief, A. M. Targeted synthesis of two iron (III) tetradentate dibasic chelating Schiff base complexes towards inhibition of acidic induced steel corrosion: Empirical and DFT insights. Appl Organomet Chem, **2022**, e6718. https://doi.org/10.1002/aoc.6718
